# Neutralising antibody responses against SARS-CoV-2 Omicron BA.4/5 and wild-type virus in patients with inflammatory bowel disease following three doses of COVID-19 vaccine (VIP): a prospective, multicentre, cohort study

**DOI:** 10.1016/j.eclinm.2023.102249

**Published:** 2023-10-05

**Authors:** Zhigang Liu, James L. Alexander, Kaixing Le, Xin Zhou, Hajir Ibraheim, Sulak Anandabaskaran, Aamir Saifuddin, Kathy Weitung Lin, Leon R. McFarlane, Laura Constable, Rocio Castro Seoane, Nikhil Anand, Claire Bewshea, Rachel Nice, Andrea D'Mello, Gareth R. Jones, Sharmili Balarajah, Francesca Fiorentino, Shaji Sebastian, Peter M. Irving, Lucy C. Hicks, Horace RT. Williams, Alexandra J. Kent, Rachel Linger, Miles Parkes, Klaartje Kok, Kamal V. Patel, Julian P. Teare, Daniel M. Altmann, Rosemary J. Boyton, Ailsa L. Hart, Charlie W. Lees, James R. Goodhand, Nicholas A. Kennedy, Katrina M. Pollock, Tariq Ahmad, Nick Powell, VIP study investigators, Ijeoma Chukwurah, Sulaimaan Haq, Jonathan Lo, Parita Shah, Stephanie Wilken-Smith, Anitha Ramanathan, Mikin Patel, Lidia Romanczuk, Rebecca King, Jason Domingo, Djamila Shamtally, Vivien Mendoza, Joanne Sanchez, Hannah Stark, Bridget Knight, Louise Bee, Charmaine Estember, Anna Barnes, Darcy Watkins, Sam Stone, John Kirkwood, Marian Parkinson, Helen Gardner-Thorpe, Kate Covil, Lauranne Derikx, Beatriz Gros Alcalde, Irish Lee, Bessie Cipriano, Giuseppe Ruocco, Manisha Baden, Graham Cooke, Evgenia Kourampa, Ciro Pasquale, Elena Robisco-Diaz, Suhaylah Bhatti

**Affiliations:** a Department of Metabolism, Digestion and Reproduction, Imperial College London, London, UK; b Department of Gastroenterology, Imperial College Healthcare NHS Trust, London, UK; c Department of Gastroenterology, St Marks Hospital and Academic Institute, Gastroenterology, London, UK; d Department of Infectious Disease, Imperial College London, London, UK; e Exeter Inflammatory Bowel Disease and Pharmacogenetics Research Group, University of Exeter, Exeter, UK; f Department of Clinical Chemistry, Exeter Clinical Laboratory International, Royal Devon University Healthcare NHS Foundation Trust, Exeter, UK; g Division of Medicine & Integrated Care, Imperial College Healthcare NHS Trust, London, UK; h Department of Gastroenterology, Western General Hospital, NHS Lothian, Edinburgh, UK; i Centre for Inflammation Research, The Queen’s Medical Research Institute, The University of Edinburgh, Edinburgh, UK; j Department of Surgery and Cancer, Imperial College London, London, UK; k Nightingale-Saunders Clinical Trials & Epidemiology Unit (King’s Clinical Trials Unit), King’s College London, London, UK; l Department of Gastroenterology, Hull University Teaching Hospitals NHS Trust, Hull, UK; m Hull York Medical School, University of Hull, Hull, UK; n Department of Gastroenterology, Guy's and St Thomas' NHS Foundation Trust, London, UK; o School of Immunology & Microbial Sciences, King's College London, London, UK; p Department of Gastroenterology, King's College Hospital, London, UK; q The NIHR Bioresource, University of Cambridge, Cambridge, UK; r Department of Gastroenterology, Cambridge University Hospitals NHS Trust, Cambridge, UK; s Department of Gastroenterology, Bart's Health NHS Trust, London, UK; t Department of Gastroenterology, St George's Hospital NHS Trust, London, UK; u Department of Immunology and Inflammation, Imperial College London, London, UK; v Lung Division, Royal Brompton and Harefield Hospitals, Guy’s and St Thomas’ NHS Foundation Trust, London, UK; w Department of Gastroenterology, Royal Devon University Healthcare NHS Foundation Trust, Exeter, UK; x NIHR Imperial Clinical Research Facility and NIHR Imperial Biomedical Research Centre, London, UK

**Keywords:** SARS-CoV-2, Inflammatory bowel disease, Infliximab, Tofacitinib, Breakthrough infection

## Abstract

**Background:**

Patients with inflammatory bowel disease (IBD) receiving anti-TNF and JAK-inhibitor therapy have attenuated responses to COVID-19 vaccination. We aimed to determine how IBD treatments affect neutralising antibody responses against the Omicron BA.4/5 variant.

**Methods:**

In this multicentre cohort study, we prospectively recruited 340 adults (69 healthy controls and 271 IBD) at nine UK hospitals between May 28, 2021 and March 29, 2022. The IBD study population was established (>12 weeks therapy) on either thiopurine (n = 63), infliximab (n = 45), thiopurine and infliximab combination therapy (n = 48), ustekinumab (n = 45), vedolizumab (n = 46) or tofacitinib (n = 24). Patients were excluded if they were being treated with any other immunosuppressive therapies. Participants had two doses of either ChAdOx1 nCoV-19 or BNT162b2 vaccines, followed by a third dose of either BNT162b2 or mRNA1273. Pseudo-neutralisation assays against SARS-CoV-2 wild-type and BA.4/5 were performed. The half maximal inhibitory concentration (NT50) of participant sera was calculated. The primary outcome was anti-SARS-CoV-2 neutralising response against wild-type virus and Omicron BA.4/5 variant after the second and third doses of anti-SARS-CoV-2 vaccine, stratified by immunosuppressive therapy, adjusting for prior infection, vaccine type, age, and interval between vaccination and blood collection. This study is registered with ISRCTN (No. 13495664).

**Findings:**

Both heterologous (first two doses adenovirus vaccine, third dose mRNA vaccine) and homologous (three doses mRNA vaccine) vaccination strategies significantly increased neutralising titres against both wild-type SARS-CoV-2 virus and the Omicron BA.4/5 variant in healthy participants and patients with IBD. Antibody titres against BA.4/5 were significantly lower than antibodies against wild-type virus in both healthy participants and patients with IBD (p < 0.0001). Multivariable models demonstrated that neutralising antibodies against BA.4/5 after three doses of vaccine were significantly lower in patients with IBD on infliximab (Geometric Mean Ratio (GMR) 0.19 [0.10, 0.36], p < 0.0001), infliximab and thiopurine combination (GMR 0.25 [0.13, 0.49], p < 0.0001) or tofacitinib (GMR 0.43 [0.20, 0.91], p = 0.028), but not in patients on thiopurine monotherapy, ustekinumab, or vedolizumab. Breakthrough infection was associated with lower neutralising antibodies against wild-type (p = 0.037) and BA.4/5 (p = 0.045).

**Interpretation:**

A third dose of a COVID-19 mRNA vaccine based on the wild-type spike glycoprotein significantly boosts neutralising antibody titres in patients with IBD. However, responses are lower against the Omicron variant BA.4/5, particularly in patients taking anti-TNF and JAK-inhibitor therapy. Breakthrough infections are associated with lower neutralising antibodies and immunosuppressed patients with IBD may receive additional benefit from bivalent vaccine boosters which target Omicron variants.

**Funding:**

10.13039/100004319Pfizer.

**Research in context**

**Evidence before this study**

We searched PubMed and Embase, without language restrictions, for studies published between Jan 1, 2000 and Oct 31, 2022, investigating humoral responses to vaccination in immunosuppressed individuals. We used the search terms (“vaccine” OR “vaccination”) AND (“immunosuppression” OR “immunosuppressive” OR “immunomodulator” OR “thiopurine” OR “azathioprine” OR “biologic” OR “tumour necrosis factor” OR “infliximab” OR “ustekinumab” OR “anti-integrin” OR “vedolizumab” OR “JAK inhibitor” OR “tofacitinib”) AND (“antibody” OR “humoral” OR “immune response”) OR (“Omicron”). We have previously shown that third doses of COVID-19 vaccines boost serological responses in patients with inflammatory bowel disease (IBD) receiving six different commonly used immunosuppressive treatment regimens. However, COVID-19 vaccine-induced antibody responses are diminished relative to healthy controls in patients with IBD taking anti-TNF and JAK-inhibitor therapies, but not anti-integrin or thiopurine monotherapy, following two and three vaccine doses. Breakthrough infection is more common in patients with IBD receiving the anti-TNF therapy infliximab compared with the gut-selective anti-integrin therapy vedolizumab. We have recently shown that neutralising antibody responses against the Omicron BA.1 variant of concern are reduced in immunosuppressed patients with IBD in comparison to neutralising responses against the wild-type virus. However, there is currently scarce data on neutralising responses against the Omicron BA.4/5 variant and the related risk of infection.

**Added value of this study**

To our knowledge, this is the first study to evaluate neutralising antibodies against the Omicron BA.4/5 variant following three doses of COVID-19 vaccine in patients receiving different immunosuppressive treatments used in IBD. We show that, although all groups had a significant boost in vaccine-induced neutralising antibody responses after a third dose, levels were significantly reduced in those patients treated with infliximab or tofacitinib. Neutralising responses against BA.4/5 in patients receiving infliximab and tofacitinib were more than one order of magnitude lower than against wild-type virus and lower neutralising antibodies against BA.4/5 were associated with a higher risk of breakthrough infection.

**Implications of all the available evidence**

Our data show that a third dose of an originator COVID-19 vaccine targeting the wild-type virus spike protein boosts neutralising antibodies against the Omicron BA.4/5 variant in immunosuppressed patients with IBD, but responses against BA.4/5 are significantly lower irrespective of immunosuppressive treatment. Combined with evidence that previous SARS-CoV-2 infection further augments humoral responses to vaccination, these results support the rollout of booster doses in immunosuppressed patients with IBD. In the context of emerging variants of concern, and evidence that patients treated with anti-TNF are at higher risk of breakthrough infection, our data also support the prioritisation of future booster dosing to those with diminished responses to vaccination, including patients taking anti-TNF or tofacitinib.

## Introduction

Vaccination is an effective population strategy for preventing severe COVID-19 disease and death as well as reducing rates of SARS-CoV-2 infection.^1^, ^2^, ^3^, ^4^, ^5^ However, since vaccines are developed mainly from trials recruiting healthy people, data on vaccine efficacy in immunosuppressed populations, including patients with inflammatory bowel disease (IBD) taking immunosuppressive drugs, are lacking. The emergence of new variants, particularly more transmissible strains, such as the Omicron variants, has resulted in high infection rates in unvaccinated and vaccinated individuals.^6^ Specifically, due to the L452R and F486V mutations in the spike protein, the BA.4/5 variant displayed reduced neutralisation by the serum from individuals vaccinated with three doses of vaccine compared with precedent Omicron variants.^7^ Concerns about more transmissible variants are especially pertinent for immunosuppressed individuals, who are more prone to infection, viral persistence and COVID-19 disease or have impaired immune responses to vaccination.^8^ Viral infection and persistence constitute a threat, both to patient health and pandemic control, with the risk of disease, onward transmission and evolution of new variants of concern (VOC). We, and others, have shown that patients with IBD established on immunosuppressive drugs, including infliximab (anti-tumour necrosis factor, or anti-TNF monoclonal antibody) or tofacitinib (a pan Janus kinase, or JAK inhibitor), have significantly lower vaccine-induced antibody responses following two and three doses of COVID-19 vaccination compared to controls.^9^, ^10^, ^11^, ^12^ Anti-TNF treatment is also associated with accelerated loss of circulating vaccine-induced antibodies and anti-TNF treated patients are more likely to develop breakthrough infections after two doses of vaccine.^8^^,^^12^^,^^13^

In some countries, including the UK, patients treated with immunosuppressant drugs, including patients with IBD are prioritised to receive a third primary and booster vaccine dose.^14^ The British Society of Gastroenterology advises that a third dose of a SARS-CoV-2 vaccine should be offered no earlier than 8 weeks after the second dose to all patients with inflammatory bowel disease who are 12 years and over and who are receiving any immunosuppressive treatment. There is no antibody testing prior to vaccination and few results or outcomes have been reported following the third dose of vaccine in patients with IBD. In addition, heterologous vaccination regimens (e.g. two doses of adenovirus vector vaccine followed by one dose of mRNA vaccine) have been used in many countries and were reported to be effective in healthy individuals.^15^ Given the continuing emergence of Omicron variants, further studies are needed to understand how key immunosuppressive drug regimens impact vaccine-induced immunogenicity against the more transmissible variant. This study aimed to evaluate the functional neutralising responses directed against BA.4/5 in an immunosuppressed population of patients with IBD.

## Methods

### Study design and participants

**Fig. 1:**
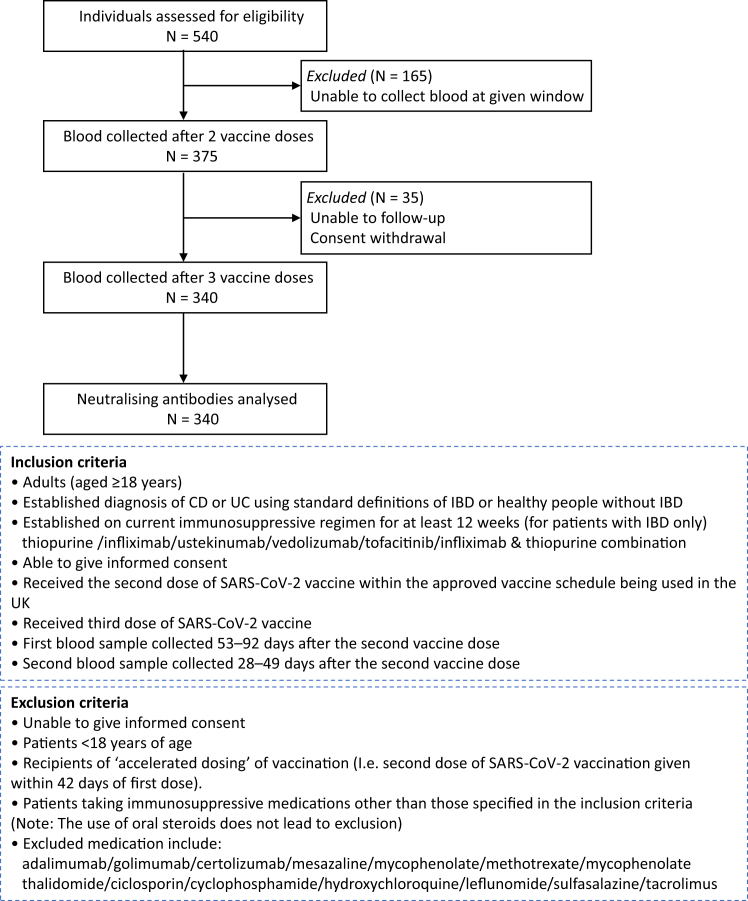
Flow diagram illustrating the study design and participant selection. NT50: 50% neutralisation titre. IBD: inflammatory bowel disease. UC: ulcerative colitis. CD: Crohn’s disease.

VIP (SARS-CoV-2 Vaccination Immunogenicity in Immunosuppressed inflammatory bowel disease Patients) is a UK multi-centre prospective observational study aimed to evaluate the immunogenicity of COVID-19 vaccination in patients with IBD on six different immunosuppressive treatment regimens (infliximab, thiopurine, infliximab and thiopurine combination therapy, ustekinumab, vedolizumab or tofacitinib). This study adheres to the STROBE guidelines. Participant recruitment, inclusion and exclusion criteria have been described previously.^9^ Blood was collected from participants 53–92 days after the second vaccine dose and 28–49 days after the third vaccine dose (Fig. 1). Participants either received homologous (3 doses of mRNA vaccine) or heterologous (2 doses of adenovirus vector followed by one dose of mRNA vaccine) vaccination schedules.

Participants were included after providing informed, written consent. The Wales Research Ethics Committee 5 approved the study (REC reference 21/WA/0105) in March, 2021. The study was registered with the ISRCTN (No: 13495664) registry.

### Anti-SARS-CoV-2 antibody electrochemiluminescence assay

The Roche Elecsys anti-SARS-CoV-2 (N) immunoassay is a sandwich electrochemiluminescence immunoassay that employs a recombinant protein of the nucleocapsid antigen for the determination of antibodies against SARS-CoV-2 infection. The manufacturer reports clinical sensitivity and specificity of 99.5% and 99.8%, respectively, >14 days after PCR-confirmed COVID-19 using a cut-off index (COI) of 1. It is reported that anti-N antibody responses following SARS-CoV-2 natural infection are impaired in patients treated with immunosuppressant drugs such as infliximab.^8^^,^^16^ Results showed that a threshold of 0.12 times the cut-off index provides 100% specificity for determining prior SARS-CoV-2 infection.^12^ Therefore, in the current study, anti-N greater than or equal to 0.12 was deemed to indicate prior SARS-CoV-2 infection.

### Pseudo neutralisation assay

The SARS-CoV-2 neutralisation assays were conducted using pseudo-typed viruses (PSV). Pseudo-typed SARS-CoV-2 lentiviruses were produced in HEK293T cells using a SARS-CoV-2 spike plasmid (wild-type strain or BA.4/5), HIV-1 gag-pol plasmid and a firefly luciferase reporter.^17^ Participant sera were serially diluted and incubated with PSV viral supernatant for 1 h. HEK293T-ACE2 cells were then co-incubated with the sera and PSV for 72 h before measurement of the luciferase activity using the Bright-Glo Luciferase assay system (Promega, Madison, WI). NT50 neutralisation titres were calculated as the dilution at which relative luminescence was reduced by 50% compared with control.

### Outcome measures

Our primary outcome was anti-SARS-CoV-2 neutralising response against wild-type virus and the Omicron BA.4/5 variant after the second and third doses of anti-SARS-CoV-2 vaccine, stratified by baseline immunosuppressive therapy, adjusting for prior infection, vaccine type, age, and the interval between vaccination and blood sampling.

The secondary outcome was risk of breakthrough infection after two doses of vaccination. SARS-CoV-2 infection was defined by participants who reported a PCR or lateral flow test confirming SARS-CoV-2 infection and/or a concentration of Roche Elecsys anti-SARS-CoV-2 nucleocapsid immunoassay nucleocapsid antibodies in the serum above 0.11 U/mL.^9^^,^^12^ The data collection range was between 28th May 2021 and 29th March 2022.

### Variables

Demographics were recorded as variables: age, gender, ethnicity, comorbidities, height and weight, smoking status, postcode, IBD disease activity (defined by patient-reported outcomes [PRO2]),^18^^,^^19^ SARS-CoV-2 symptoms aligned to the COVID-19 symptoms study (symptoms, previous testing and hospital admissions for COVID-19), SARS-CoV-2 test date and results, vaccine schedules (type and date of each vaccination) and date of blood collection. Data were entered electronically into a purpose-designed REDCap database hosted at the Royal Devon University Healthcare NHS Foundation Trust.^20^ Participants without access to the internet or electronic devices completed their questionnaires on paper case record forms that were subsequently entered by local research teams.

### Statistical analysis

A statistical analysis plan was approved by the Study Management. Analyses were undertaken using R 4.1.0 (R Foundation for Statistical Computing, Vienna, Austria). p values < 0.05 with two-tailed tests were considered significant. Antibody concentrations are reported as geometric mean and standard deviation. Other continuous data are reported as a median and interquartile range, and discrete data as numbers and percentages, unless otherwise stated. The comparison of breakthrough infection rates in patients with IBD above and below NT50 cut-off was performed with Fisher’s exact test. To determine the NT50 cut-off value, we initially consulted relevant literature to understand commonly used cut-off values. However, due to methodological differences across studies, these values were not directly comparable. Therefore, we conducted our own data analysis, comparing breakthrough infection rates at various NT50 levels. This allowed us to identify a cut-off value that was most effective in differentiating breakthrough infection rates in our specific study population.

Multivariable linear regression models were used to identify factors independently associated with neutralising antibody concentrations. These variables were initially tested in the regression models: age, gender, ethnicity, body mass index, height, weight, smoking, IBD subtype, first two doses of vaccine type (mRNA vs adenovirus vector vaccine), homologous (3 doses of mRNA) or heterologous (2 doses of adenovirus vector followed by 1 dose of mRNA vaccine) vaccination schedules, interval days between the second dose of vaccine and first blood sampling, interval days between the third dose of vaccine and second blood sampling, interval days between the first and second dose of vaccination, and interval days between the second and third dose of vaccination. We used backward stepwise regression and calculated the Akaike Information Criterion (AIC), which takes into account both the goodness of fit of the model and the likelihood of overfitting. In this approach, variables are iteratively removed from the model in a way that results in the largest decrease in AIC. Based on their clinical significance, and for consistency across all models including those after two and three vaccine doses and against both the wild-type and Omicron variants, we included the following variables in the final models: age, prior infection, interval in weeks from vaccination to blood sampling, and mRNA vaccination (or homologous vaccination for the third vaccine dose). In the regression model for the third dose, we also included NT50 after two vaccine doses as a predictor, since it could influence the NT50 after three doses. Results are presented after exponentiation so that the model's coefficients correspond to the geometric mean ratio associated with each covariate. The linearity, homogeneity of variance, collinearity, influential observations, and normality of residuals of each multivariate model were assessed using the performance package in R. Linearity assumption for key continuous variables including age, NT50 after second dose and time from second or third dose to sampling were also assessed individually with partial regression plots using the car package in R.

We also performed sensitivity analyses to investigate how IBD affect the neutralising antibodies. We excluded healthy controls and performed multivariable regression analysis to verify the effect of IBD drugs on neutralising antibodies. Since vaccination, neutralising antibodies and prior infection are interrelated, we also carried out multivariable regression analysis excluding participants with prior infection.

### Role of the funding source

The funder of the study had no role in study design, data collection, data analysis, data interpretation, or writing of the report. ZL, JLA and NP had access to dataset and had final responsibility for the decision to submit for publication.

## Results

Between 28th May 2021 and 29th March 2022, we recruited 271 participants with IBD established on key immunosuppressive treatment regimens, including infliximab (n = 45), thiopurines (n = 63), infliximab and thiopurine combination therapy (n = 48), ustekinumab (n = 45), vedolizumab (n = 46) and tofacitinib (n = 24). We additionally recruited a population of healthy, non-IBD control individuals (n = 69). Participants had received either two doses of mRNA vaccine (BNT162b2) or adenovirus vector vaccine (ChAdOx1 nCoV-19) as their primary vaccination schedule and received mRNA vaccine (BNT162b2 or mRNA-1273) as the third dose. Participant characteristics are shown in Table 1.

**Table 1:** Demographics of the cohort in this study.

Characteristics	Control (N = 69, 20%)	Infliximab (N = 45, 13%)	Infliximab + Thiopurine (N = 48, 14%)	Thiopurine (N = 63, 19%)	Tofacitinib (N = 24, 7.1%)	Ustekinumab (N = 45, 13%)	Vedolizumab (N = 46, 14%)	p-value
Age	36.40 (28.70, 52.10)	47.20 (35.50, 56.50)	39.20 (31.03, 51.27)	44.10 (33.65, 55.15)	46.00 (36.33, 54.68)	41.80 (32.80, 54.00)	45.50 (37.45, 61.57)	0.043
Gender								0.0040
Female	45 (66.18%)	22 (48.89%)	22 (45.83%)	34 (54.84%)	8 (33.33%)	25 (55.56%)	12 (28.57%)	
Male	23 (33.82%)	23 (51.11%)	26 (54.17%)	28 (45.16%)	16 (66.67%)	20 (44.44%)	30 (71.43%)	
Ethnicity								0.80
Non-white	10 (14.71%)	8 (17.78%)	8 (16.67%)	12 (19.35%)	4 (16.67%)	6 (13.33%)	11 (26.19%)	
White	58 (85.29%)	37 (82.22%)	40 (83.33%)	50 (80.65%)	20 (83.33%)	39 (86.67%)	31 (73.81%)	
BMI	23.11 (21.42, 25.57)	25.23 (23.31, 28.47)	24.91 (22.49, 27.11)	24.01 (21.73, 26.79)	25.26 (22.81, 28.94)	25.15 (22.54, 29.14)	24.76 (23.15, 28.16)	0.026
Vaccine (first two doses)								
ChAdOx1 nCoV-19	31 (44.93%)	20 (44.44%)	34 (70.83%)	38 (60.32%)	16 (66.67%)	30 (66.67%)	27 (58.70%)	0.013
mRNA-1273	2 (2.90%)	0	0	0	1 (4.17%)	0	0	
BNT162b2	35 (50.72%)	25 (55.56%)	14 (29.17%)	24 (38.10%)	7 (29.17%)	15 (33.33%)	15 (32.61%)	
Unknown	1 (1.45%)	0	0	1 (1.59%)	0	0	4 (8.70%)	
Vaccine (3rd dose)								<0.001
mRNA-1273	26 (37.68%)	3 (6.67%)	4 (8.33%)	4 (6.35%)	3 (12.50%)	1 (2.22%)	2 (4.35%)	
BNT162b2	37 (53.62%)	26 (57.78%)	30 (62.50%)	36 (57.14%)	20 (83.33%)	36 (80%)	28 (60.87%)	
Unknown	6 (8.70%)	16 (35.56%)	14 (29.17%)	23 (36.51%)	1 (4.17%)	8 (17.78%)	16 (34.78%)	
Diagnosis								<0.001
Crohn’s disease	–	30 (66.67%)	30 (62.50%)	27 (42.86%)	1 (4.17%)	44 (97.78%)	18 (39.13%)	
Unknown IBD	–	2 (4.44%)	2 (4.17%)	1 (1.59%)	0	0	1 (2.17%)	
Ulcerative colitis	–	13 (28.89%)	16 (33.33%)	35 (55.56%)	23 (95.83%)	1 (2.22%)	27 (58.70%)	
Smoking								0.086
Currently	1 (1.47%)	2 (4.44%)	3 (6.25%)	1 (1.61%)	2 (8.33%)	3 (6.67%)	5 (11.90%)	
Not currently	16 (23.53%)	12 (26.67%)	15 (31.25%)	21 (33.87%)	13 (54.17%)	14 (31.11%)	12 (28.57%)	
Never	51 (75%)	31 (68.89%)	30 (62.50%)	40 (64.52%)	9 (37.50%)	28 (62.22%)	25 (59.52%)	
Breakthrough infection	12 (17.39%)	6 (13.33%)	12 (25.00%)	12 (19.05%)	3 (12.50%)	8 (17.78%)	5 (10.87%)	0.66
Interval days: 2nd vaccine dose to 1st sampling	80 (77.75, 86)	74.50 (61, 87)	84 (62, 88)	76 (62.50, 85)	79 (63, 87.50)	82.50 (65.75, 88)	79 (64, 87)	0.36
Interval days: 3rd vaccine dose to 2nd sampling	37 (34, 42)	40 (32, 45.75)	40 (37.50, 46.25)	40.50 (32, 43)	35.50 (32, 41.75)	40.50 (33.75, 45)	40 (33.50, 42)	0.32
Interval days: 1st to 2nd vaccine dose	64 (57, 77)	74.50 (69, 77)	77 (76, 79)	77 (75, 79)	72 (60.50, 76.50)	76 (70, 79)	77 (71, 80)	<0.001
Interval days: 2nd to 3rd vaccine dose	174 (151.50, 184.50)	179.50 (167.75, 188.50)	171 (152.50, 184.50)	176 (163, 187)	175 (152, 182)	177 (156, 188.50)	186 (166.75, 194.50)	0.33
Heart disease	0	1 (2.22%)	0	0	0	0	3 (7.14%)	0.0090
Lung disease	4 (5.88%)	7 (15.56%)	6 (12.50%)	6 (9.68%)	3 (12.50%)	4 (8.89%)	3 (7.14%)	0.68
Kidney disease	0	2 (4.44%)	0	1 (1.61%)	0	1 (2.22%)	1 (2.38%)	0.47
Diabetes	1 (1.47%)	3 (6.67%)	0	4 (6.45%)	0	3 (6.67%)	3 (7.14%)	0.22
Cancer	0	1 (2.22%)	0	1 (1.61%)	0	0	1 (2.38%)	0.61

Continuous variables were presented as median (IQR). Other variables were presented as percentages within each group. Kruskal–Wallis H test (for continuous variables) and Fisher's exact test (for categorical variables) were employed to test the significance.

BMI: body mass index.

**Fig. 2:**
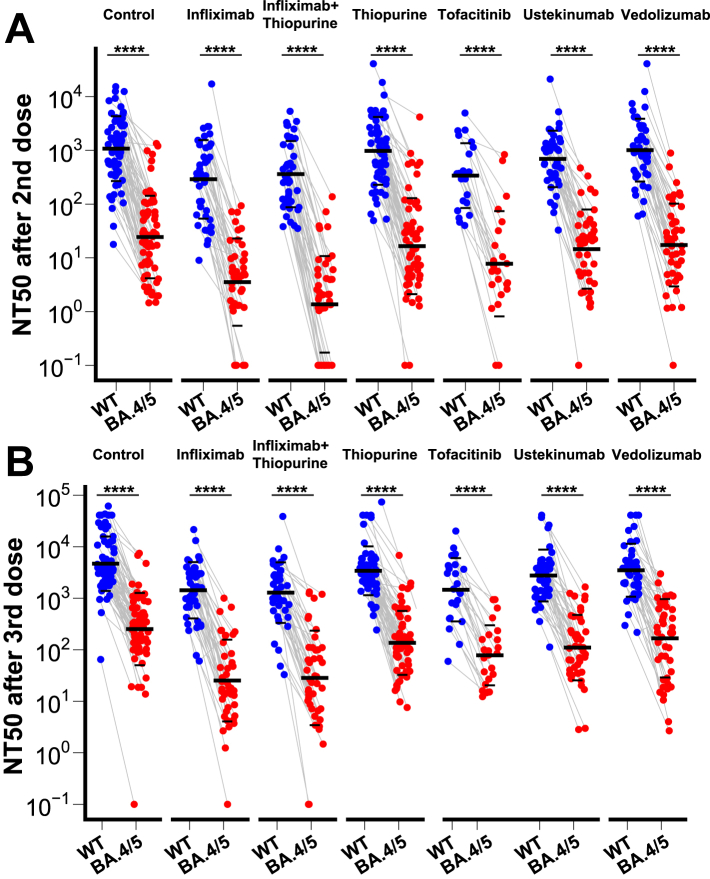
NT50 against SARS-CoV-2 wild-type (WT) and BA.4/5 in patients with IBD treated with different immunosuppressive medications and healthy controls after two (A) or three (B) doses of vaccine. The crossbar represented the geometric mean and SD. Wilcoxon signed-rank test was performed to test the significance. Samples unable to inhibit half of the virus infection were plotted with the NT50 equal to 0.1.

**Fig. 3:**
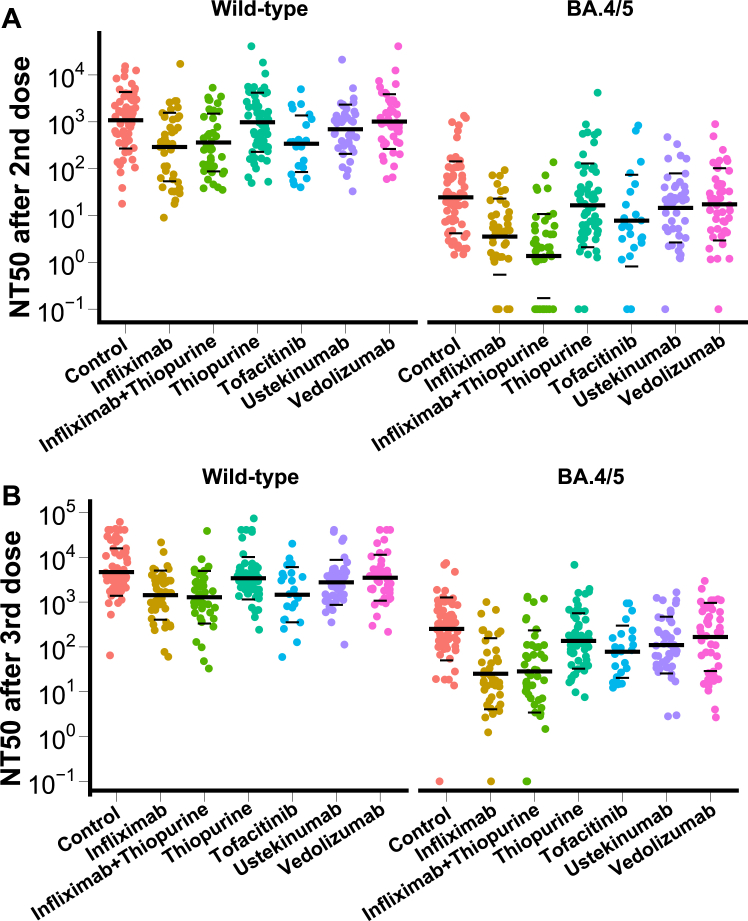
NT50 in patients with IBD treated with different immunosuppressive medications and healthy controls against SARS-CoV-2 wild-type (WT) and BA.4/5 after two (A) or three (B) doses of vaccine. The crossbar represented the geometric mean and SD. Samples unable to inhibit half of the virus infection were plotted with the NT50 equal to 0.1.

To evaluate vaccine-induced humoral responses we employed a pseudo neutralisation assay against the SARS-CoV-2 wild-type (WT) and Omicron BA.4/5 variant. After two or three doses of vaccine, 50% neutralisation titres (NT50) against Omicron BA.4/5 were significantly lower compared to against WT both in healthy controls and all six IBD patient groups (p values < 0.0001 comparing NT50 against WT and BA.4/5 in each group. Fig. 2, Table 2). We next evaluated the effect of a third vaccine dose compared to two doses of vaccine. Reassuringly, neutralising titres against WT and BA.4/5 significantly increased after a third dose of vaccine compared to titres after a second dose of vaccine in all treatment groups (Supplementary Fig. S1 p < 0.0001 comparing NT50 after 2 and 3 vaccine doses in each group). We then plotted the NT50 between healthy controls and patients with IBD with different treatments. Patients treated with infliximab, infliximab plus thiopurine combination therapy, or tofacitinib showed lower NT50 against WT and BA.4/5 both after 2 and 3 vaccine doses compared to healthy controls (Fig. 3). 27 patients did not generate an NT50 against the BA.4/5 variant after two doses of vaccine, 21 of whom received infliximab treatment, comprising 30% patients of all patients treated with infliximab (Fig. 3A). After 3 doses of vaccine, three patients treated with infliximab still did not generate an NT50 against BA.4/5 (Fig. 3B).

**Table 2:** 50% neutralising titres and confidence intervals against SARS-CoV-2 wild-type strain and Omicron BA.4/5, by participant groups and SARS-CoV-2 vaccination doses.

	NT50 against wild-type after 2 vaccine doses	NT50 against BA.4/5 after 2 vaccine doses	NT50 against wild-type after 3 vaccine doses	NT50 against BA.4/5 after 3 vaccine doses
Control	1077 [769.9, 1507]	24.38 [15.90, 37.40]	4680 [3486, 6284]	252.0 [170.4, 372.6]
Infliximab	289.6 [172.5, 486.1]	3.539 [1.990, 6.292]	1430 [970.5, 2107]	25.22 [14.37, 44.27]
Infliximab + Thiopurine	361.5 [237.3, 550.8]	1.363 [0.7379, 2.517]	1284 [855.4, 1927]	28.37 [15.05, 53.50]
Thiopurine	976.2 [670.7, 1421]	16.50 [9.698, 28.08]	3410 [2578, 4509]	136.0 [94.36, 196.0]
Tofacitinib	339.6 [186.2, 619.3]	7.762 [2.930, 20.56]	1462 [803.6, 2658]	78.15 [44.32, 137.8]
Ustekinumab	693.6 [478.0, 1007]	14.52 [8.616, 24.48]	2759 [1941, 3923]	110.0 [70.57, 171.4]
Vedolizumab	1011 [677.8, 1508]	17.31 [10.22, 29.31]	3496 [2462, 4964]	166.9 [99.21, 280.8]

We performed multivariable linear regression to determine whether other variables impacted vaccine-induced immune responses. We first carried out model diagnostic checks to confirm the linear regression models perform well (Supplementary Figs. S2–S5). The model-predicted lines match the observed data in the posterior predictive check. Both linearity and homogeneity of variance checks show nearly flat and horizontal reference lines, confirming their assumptions are met. All points are inside the contour lines in the influential observations check, indicating no overly influential observations. The collinearity assessment indicates that the variance inflation factor (VIF) for all models falls below 2, signifying that multicollinearity is unlikely to be an issue. The normality of residuals check confirms that residuals are approximately normally distributed. Linearity assumption for key continuous variables such as age, NT50 after second dose and time from second or third dose to sampling were assessed by partial regression plots (Supplementary Figs. S6 and S7), which demonstrated that the linearity assumption is likely met. Overall, the models are well-specified and fit the data well.

**Fig. 4:**
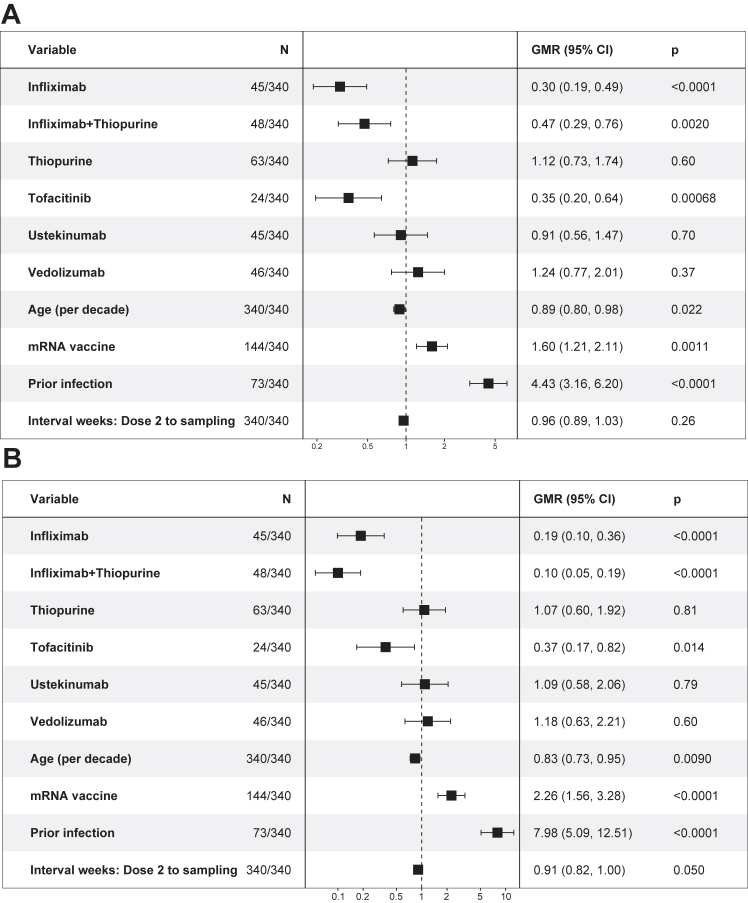
Multivariable regression model, adjusted for age, prior infection, first two doses of vaccine type (mRNA vs adenovirus vector vaccine) and interval between second vaccine dose and blood sampling, showing the exponentiated coefficients of linear regression models of log-transformed NT50 after two doses stratified by study treatment group. A: NT50 against wild-type. B: NT50 against BA.4/5. The values represent the geometric mean ratios of NT50 associated with each variable.

**Fig. 5:**
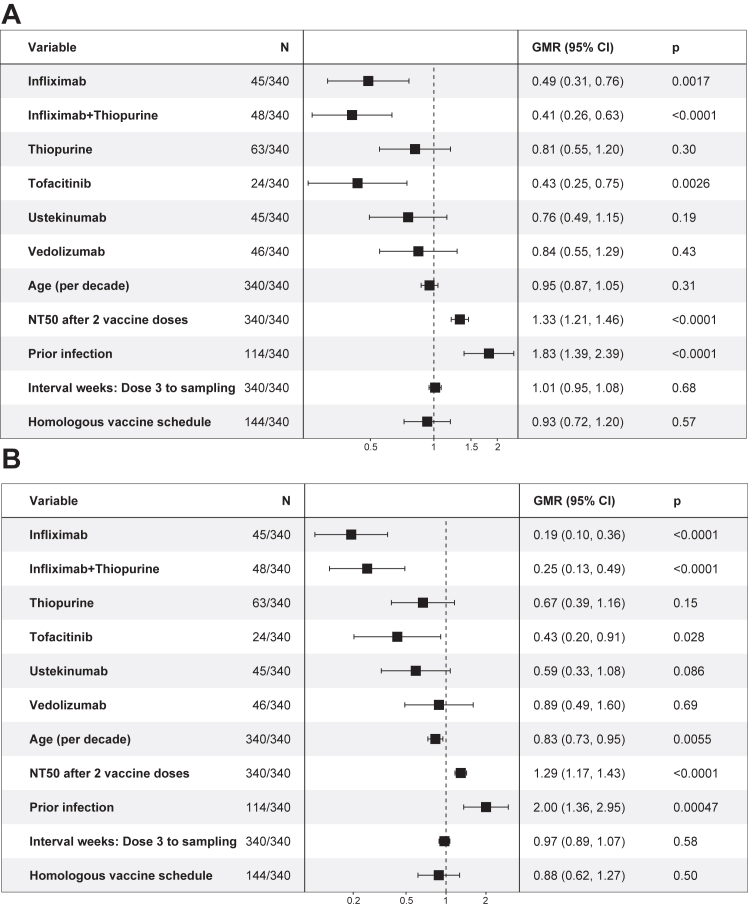
Multivariable regression model showing the exponentiated coefficients of linear regression models of log-transformed NT50 after three doses stratified by study treatment group. A: NT50 against wild-type. B: NT50 against BA.4/5. The values represent the geometric mean ratios of NT50 associated with each variable.

In the regression results, lower neutralising antibody titres against the WT virus and the BA.4/5 variant were observed in patients with IBD established on infliximab monotherapy (all comparisons p < 0.01), infliximab and thiopurine combination therapy (all comparisons p < 0.01) or tofacitinib (all comparisons p < 0.05), after two or three doses of COVID-19 vaccine (Fig. 4, Fig. 5). Prior infection was independently associated with higher NT50 (all comparisons p < 0.001). Older age was associated with lower NT50 against WT after two doses (p = 0.022), against BA.4/5 after two doses (p = 0.0090) and three doses (p = 0.0055) of COVID-19 vaccine. Two doses of mRNA vaccine induced higher titres of neutralising antibodies than two doses of adenovirus vector vaccine (both p < 0.01). All patients received mRNA vaccine as the third dose, and there was no significant difference in the neutralising titres after three doses between homologous and heterologous booster schedules (both p > 0.05).

To investigate variations in neutralising antibody levels among patients with IBD treated with different drugs, we conducted a multivariable regression analysis excluding healthy controls (Supplementary Figs. S8 and S9). In light of both our study and the CLARITY study [21] indicating that vedolizumab does not impair antibody responses, we used vedolizumab-treated patients as the reference group. The results, which excluded healthy controls, were consistent with our original findings that included healthy controls, demonstrating that treatment with infliximab, the combination of infliximab and thiopurine, and tofacitinib resulted in lower neutralising antibody responses.

We also performed a sub-analysis excluding participants with prior infections (Supplementary Figs. S10 and S11). The neutralising antibodies were lower in patients treated with infliximab, the combination of infliximab and thiopurine, and tofacitinib after two vaccine doses (all p < 0.05). As time went on, more people contracted the infection, so more participants were excluded after the third vaccine dose. With a smaller sample size, some comparisons became non-significant, but the trend remained the same. After three doses of vaccination, lower neutralising antibodies were found in patients treated with infliximab (p = 0.10 for wild-type, p = 0.012 for BA.4/5), a combination of infliximab and thiopurine (p = 0.0039 for wild-type, p = 0.0011 for BA.4/5), and tofacitinib (p = 0.0027 for wild-type, p = 0.13 for BA.4/5).

**Fig. 6:**
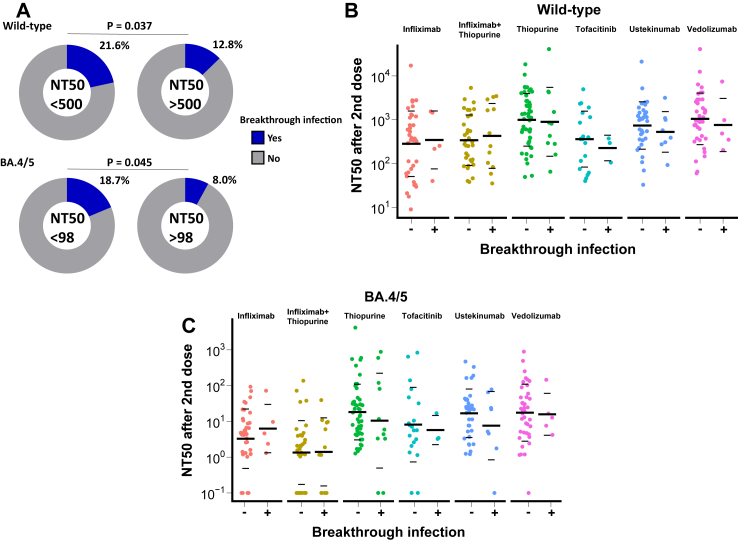
The correlation of breakthrough infection with NT50 after two vaccine doses in patients with IBD. A: The percentage of breakthrough infection above and below NT50 cut-off. Statistical difference was calculated with a Fisher’s test. B: NT50 against wild-type after two doses of vaccine stratified by treatment and breakthrough infection. C: NT50 against BA.4/5 after two doses of vaccine stratified by treatment and breakthrough infection. The crossbar represented the geometric mean and SD.

Next, we investigated the breakthrough infection rate in this cohort, which was 17% in either healthy controls or patients with IBD. Then we tested the correlation of different thresholds of neutralising titres with the breakthrough infection rate (Fig. 6A). Patients with IBD with an NT50 < 500 against the WT virus after two vaccine doses had a 1.88-fold increased risk of infection compared to patients with IBD with NT50 > 500 (p = 0.037). Patients with IBD with an NT50 < 17 against BA.4/5 after two vaccine doses had a 1.96-fold increased risk of infection compared to patients with IBD with NT50 > 17 (p = 0.045). We also stratified the distribution of NT50 in breakthrough cases by immunosuppressive treatments and compared it to the distribution in uninfected cases (Fig. 6B and C). No statistically significant difference among the groups was identified which may be attributable to small sample sizes after stratification. There were fewer healthy controls with NT50 under the cut-off and no significant associations between breakthrough infection and NT50 (above or under cut-off).

We also analysed the correlation of NT50 against wild-type and BA.4/5 (Supplementary Fig. S12). Spearman correlation analysis showed that NT50 against wild-type and BA.4/5 showed a significant correlation (R = 0.76, p < 0.0001, Supplementary Fig. S12A). Previously, we reported anti-S RBD binding antibody concentrations in this cohort.^9^^,^^10^ We tested the correlation between the binding antibodies and neutralising antibodies. Results showed a strong correlation between anti-S RBD antibody concentrations and NT50 against wild-type (R = 0.76, p < 0.0001, Supplementary Fig. S12B) and BA.4/5 (R = 0.85, p < 0.0001, Supplementary Fig. S12C).

## Discussion

In this study, we have shown that neutralising antibody responses against SARS-CoV-2 wild-type and BA.4/5 variants are significantly augmented in patients with IBD following a third dose of vaccine, irrespective of their immunosuppressive treatment regimen. However, patients treated with infliximab, infliximab and thiopurine combination therapy or tofacitinib mounted significantly lower responses relative to controls. Although first-generation vaccines improved humoral immune responses against both wild-type and Omicron BA.4/5 variants, neutralising titres elicited against the BA.4/5 variant were generally poor for all individuals, and were very low in recipients of infliximab, infliximab and thiopurine combination therapy or tofacitinib. This raises concerns about whether first-generation vaccines will be sufficient to protect against continually evolving SARS-CoV-2 variants. Accordingly, with continuing antigenic drift in evolving variants, antibodies elicited against the wild-type spike will inevitably have reduced binding and neutralising activity against these novel variants. Since many mutations exist in the Omicron spike protein, this might lead to a significant escape from immune protection elicited by COVID-19 vaccine designed against SARS-CoV-2 wild-type virus.^21^

Our data also highlight some other interesting findings relevant to the care of patients with IBD. A third dose of the mRNA vaccine significantly improved responses in patients who had previously received their first two doses with either mRNA or the ChAdOx1 nCoV-19 vaccine, showing that both homologous and heterologous boosting with mRNA vaccines is effective in patients with IBD.^22^^,^^23^ Prior infection was independently associated with higher vaccine-induced neutralising antibody responses, even after a third dose of vaccine, consistent with the likelihood that further vaccine boosters will continue to incrementally increase neutralising antibody responses, which may be especially important in patients treated with anti-TNF drugs or tofacitinib, who have lower circulating antibody levels, particularly against emerging VOCs. Lower NT50 against the BA.4/5 variant was associated with higher risk of breakthrough infection, suggesting further booster, especially the recently available bivalent vaccine which was designed based on Omicron variants should be rolled out to protect people from infection or severe diseases.^24^

This study did not explore the immunological mechanisms for reduced vaccine-induced antibody responses observed in infliximab and tofacitinib. However, both drugs may directly interfere with antibody generation through suppression of primary B cell follicle formation within germinal centres, reduced activation of antibody-secreting plasma cells and memory B cells, and inhibition of dendritic cell networks.^25^, ^26^, ^27^, ^28^ Tofacitinib is a pan-JAK inhibitor with the highest activity against JAK1 and JAK3 signalling, which play a crucial role in innate and adaptive immune function, including B-cell activity.^29^, ^30^, ^31^ For instance, IL6 and IL21, are JAK1-dependent cytokines required for optimal differentiation, survival and activation of antibody-producing B cells.^32^, ^33^, ^34^

Consistent with our findings, CLARITY-IBD found that patients with IBD treated with infliximab had lower anti-S1 or neutralising antibodies against the SARS-CoV-2 wild-type and Omicron variants compared to those treated with vedolizumab after 2 or 3 vaccine doses.^8^^,^^12^^,^^16^^,^^35^ Although this was associated with an increased risk of breakthrough infection in infliximab recipients, COVID symptoms were mild, and severe disease, including hospitalisations and deaths, was reassuringly still uncommon.^8^ Other studies have also reported reduced vaccine-induced antibody responses against the WT spike protein in anti-TNF treated patients with IBD after 2 vaccine doses.^36^, ^37^, ^38^, ^39^ Interestingly, two independent studies have observed augmented antigen-specific T-cell responses in anti-TNF treated patients with IBD following 2 doses of SARS-CoV-2 vaccine doses,^36^^,^^40^ which may be important in providing anti-viral activity despite attenuated antibody responses, and may partly explain why outcomes do not appear to be worse in anti-TNF recipients following infection.^8^

Our study has important strengths. We have taken a unique approach to evaluating antibody responses directed against the dominating variant BA.4/5 in late 2022. We have also harnessed functional neutralisation assays, rather than anti-S1 serology assays employed in most other IBD studies. We have also actively recruited patients established on the main IBD drug regimens to get a broad view of the impact of different immunosuppressive mechanisms of action on vaccine-induced immunogenicity. We also prospectively recruited a population of healthy, non-IBD controls as a critical comparison. However, we acknowledge the limitations of this study. Firstly, the sample size for some drug groups, most notably tofacitinib, was small, which might limit the robustness of our findings, although our observations were highly statistically significant. Secondly, we do not report anti-viral T-cell immune responses in the current work, although we found that T-cell responses were similar in all treatment groups, except for reduced T-cell responses in patients treated with tofacitinib.^10^ Thirdly, the prior infection history was self-reported by participants who had been confirmed with the SARS-CoV-2 PCR test, or lateral flow testing, which may be susceptible to recall bias. Finally, although our study was consistent with a signal for increased risk of breakthrough infection in patients with IBD with lower titres of neutralising antibodies, the study was underpowered to answer this question definitely, and the results should be regarded with caution.

In summary, we have shown that third dose of vaccination significantly increases neutralising antibody responses against the SARS-CoV-2 wild-type and Omicron BA.4/5 variants in both healthy people and immunosuppressed patients with IBD. With more mutations in the Spike protein and ease of immune escape, antibodies to BA.4/5 are lower compared to wild-type, indicating that as new variants and mutations emerge, further vaccination, especially with second-generation bivalent vaccines targeting Omicron, is warranted to raise the antibody levels against the virus, especially in patients treated with the anti-TNF drug infliximab and the JAK inhibitor tofacitinib.

## Contributors

Study concept and design: NP, TA and ZL.

Acquisition, analysis, or interpretation of data: ZL, KL, XZ, KWL, LRM, JLA, NA, NAK, HI, SA, AS, LC, RCS, CB, ADM, GRJ, FF, SS, PMI, LCH, HRTW, AJK, MP, KK, KVP, JPT, RN, DMA, RJB, ALH, CWL, JRG, SB, RL, KMP, TA and NP.

Directly accessed and verified the underlying data and drafting of the manuscript: ZL, JLA and NP.

Critical review of the manuscript: All authors.

## Data sharing statement

Data from this study will be made available upon publication and can be accessed under a data transfer agreement from the corresponding author. Access to the data will be granted based on a reasonable request and approval of a research proposal. Any data sharing will be facilitated through a formal data access agreement, and may be subject to additional restrictions, depending on the nature of the analysis.

## Declaration of interests

ALH reports payment or honoraria for lectures, presentations, speakers bureaus, manuscript writing, or educational events from AbbVie, Arena, Atlantic, Bristol Myers Squibb, Celltrion, Ferring, Dr Falk, Galapagos, Janssen, MSD, Napp, Pfizer, Pharmacosmos, Shire, and Takeda; participation on the Global Steering Committee for Genentech. AJK reports consulting fees from Janssen; payment or honoraria for lectures, presentations, speakers bureaus, manuscript writing, or educational events from Pfizer and Takeda; support for attending meetings or travel from Janssen, Tillotts, and Norgine; and participation in a data safety monitoring board or advisory board for AbbVie. CWL received personal consulting fees from Galapagos, AbbVie, Takeda, Pfizer, Janssen and Iterative Scopes; institutional consulting fees from Trellus Health; and support for attending meetings from Galapagos, AbbVie, Takeda, Pfizer, Janssen, GSK, Gilead, Fresenius Kabi, Ferring, and Dr Falk. GRJ received speaker fees from Takeda, Ferring, Janssen, AbbVie and Fresenius Kabi. JLA received support for attending meetings from Takeda. JRG reports grants from Roche, Biogen, Celltrion Healthcare, Takeda and Galapagos. KVP received consulting fees from AbbVie, Janssen, Galapagos and Pfizer; reports payment or honoraria for lectures, presentations, speakers bureaus, manuscript writing, or educational events from AbbVie, Dr Falk, Janssen, Galapagos, Pfizer, Takeda, Tillotts and Ferring; support for attending meetings or travel from AbbVie, Dr Falk, Janssen, Galapagos, Pfizer, Takeda, Tillotts and Ferring; and participation on a data safety monitoring board or advisory board for AbbVie, Galapagos, Pfizer and Janssen. SB received research grant from Bowel Research UK and support for attending conferences from Ferring and Dr Falk, outside the submitted work. KMP is a member of the data safety monitoring board for NCT05249829 and NCT05575492; has received a fee for speaking from Seqirus and Sanofi Pasteur, and has research funding from the Chan Zuckerberg Initiative, the MRC/UKRI, the Vaccine Task Force, and NIHR Imperial BRC outside the submitted work. KK reports payment or honoraria for lectures, presentations, speakers bureaus, manuscript writing, or educational events from Janssen and Ferring; support for attending meetings or travel from Janssen and Takeda; and participation on a data safety monitoring board or advisory board for Janssen and PredictImmune. MP received research grant from Pfizer and speaker fees from Janssen. NAK reports grants from AbbVie, Biogen, Celgene, Celltrion, Galapagos, MSD, Napp, Pfizer, Pharmacosmos, Roche, and Takeda; consulting fees from Amgen, Bristol Myers Squibb, Dr Falk, Janssen, Mylan, Pharmacosmos, Galapagos, Takeda, and Tillotts; personal fees from Allergan, Celltrion, Dr Falk, Ferring, Janssen, Pharmacosmos, Takeda, Tillotts, and Galapagos; and support for attending meetings from AbbVie, Dr Falk, Tillotts and Janssen, outside the submitted work. PMI reports grants from Celltrion, Takeda, MSD, Pfizer, and Galapagos; personal fees from Gilead, Pfizer, Galapagos, Takeda, AbbVie, Celltrion, Janssen, Bristol Myers Squibb, Lilly, and Arena; speaker fees from Pfizer, Galapagos, Takeda, AbbVie, Celltrion, Janssen, Bristol Myers Squibb, Lilly, and Arena, outside the submitted work. SS reports grants from Tillotts, Takeda, Janssen, Pfizer and AbbVie; received personal fees from AbbVie, Tillotts, Janssen, Takeda, Dr Falk, Lilly and Bristol Myers Squibb; received support for attending meetings from Janssen, AbbVie and Celltrion; and serves as a member of the data safety monitoring board for AbbVie, Janssen, Takeda, Celltrion, Lilly and Bristol Myers Squibb. TA reports grant funding from Pfizer to deliver this study; grants from Celltrion, Roche, Takeda, Biogen, and Galapagos; and honoraria for lectures from Takeda and Roche, outside the submitted work. NP is the principal investigator on the research grant from Pfizer that funded the VIP study; has received research grants from Bristol Myers Squibb, Roche, Biogen, Celltrion Healthcare, Takeda, Galapagos, CCUK, AstraZeneca, Helmsley Charitable Trust, outside the submitted work; reports personal fees from Takeda, Janssen, Pfizer, Galapagos, Bristol Myers Squibb, AbbVie, Roche, Lilly, Allergan, Celgene and AstraZeneca outside the submitted work; and has served as a speaker or advisory board member for AbbVie, Allergan, Bristol Myers Squibb, Celgene, Dr Falk, AstraZeneca, Galapagos and Vifor; and serves as a member of the data safety monitoring board for Bristol Myers Squibb and AstraZeneca. All other authors declare no competing interests.
