# Supplementary material for: Neutralising antibody responses against SARS-CoV-2 Omicron BA.4/5 and wild-type virus in patients with inflammatory bowel disease following three doses of COVID-19 vaccine (VIP): a prospective, multicentre, cohort study
Source: eClinicalMedicine. 2023 Oct 5;64:102249. doi: 10.1016/j.eclinm.2023.102249 (PMC10570718; doi:10.1016/j.eclinm.2023.102249)
Supplement: Supplementary Figs. S1–S12 and Table S1 [file mmc1.pdf]

Supplementary Information

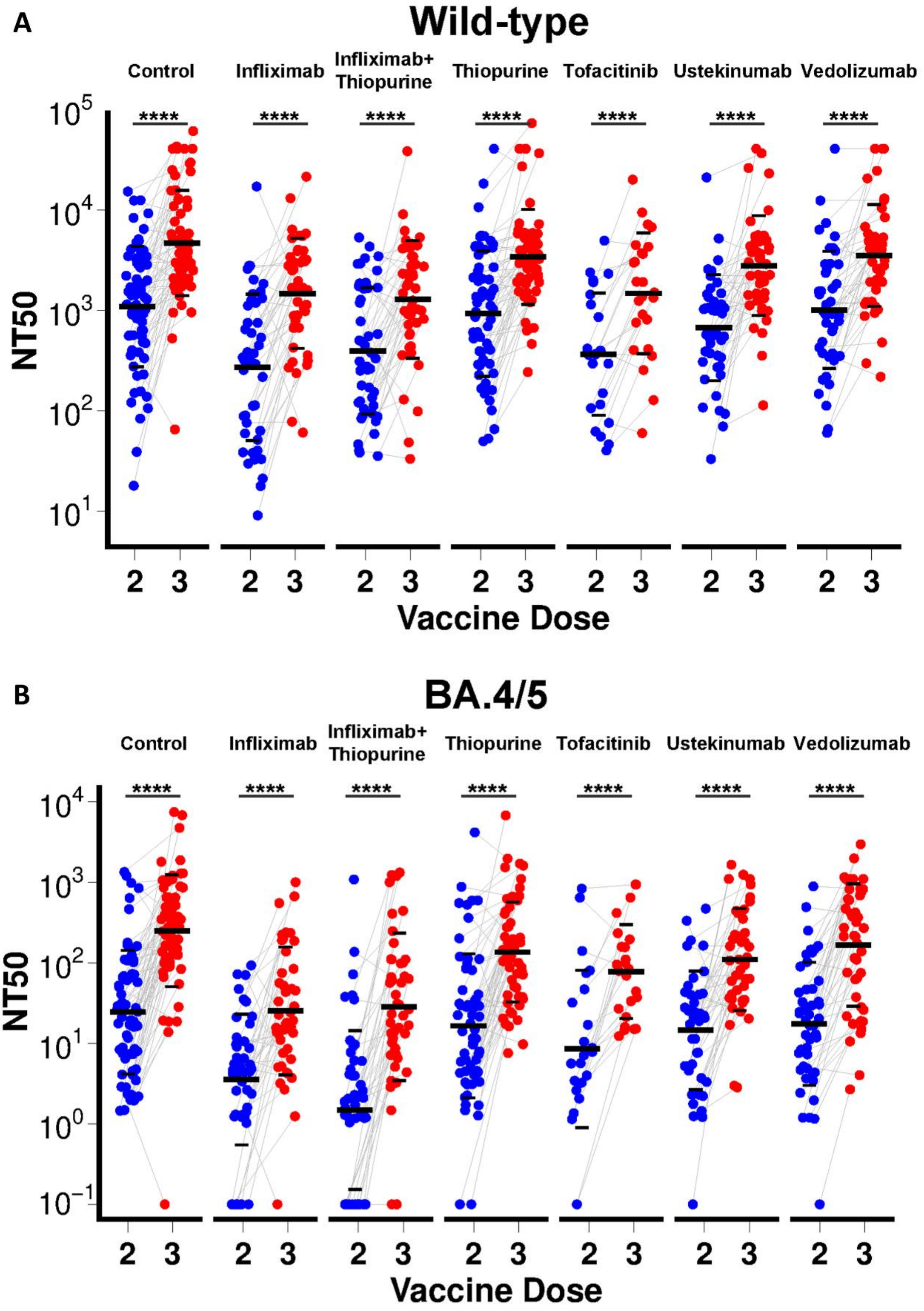

Supplementary Figure 1. Comparison of NT50 after two and three doses of vaccine against SARS-CoV-2 wild type and BA.4/5 in IBD patients treated with different medications and healthy controls.

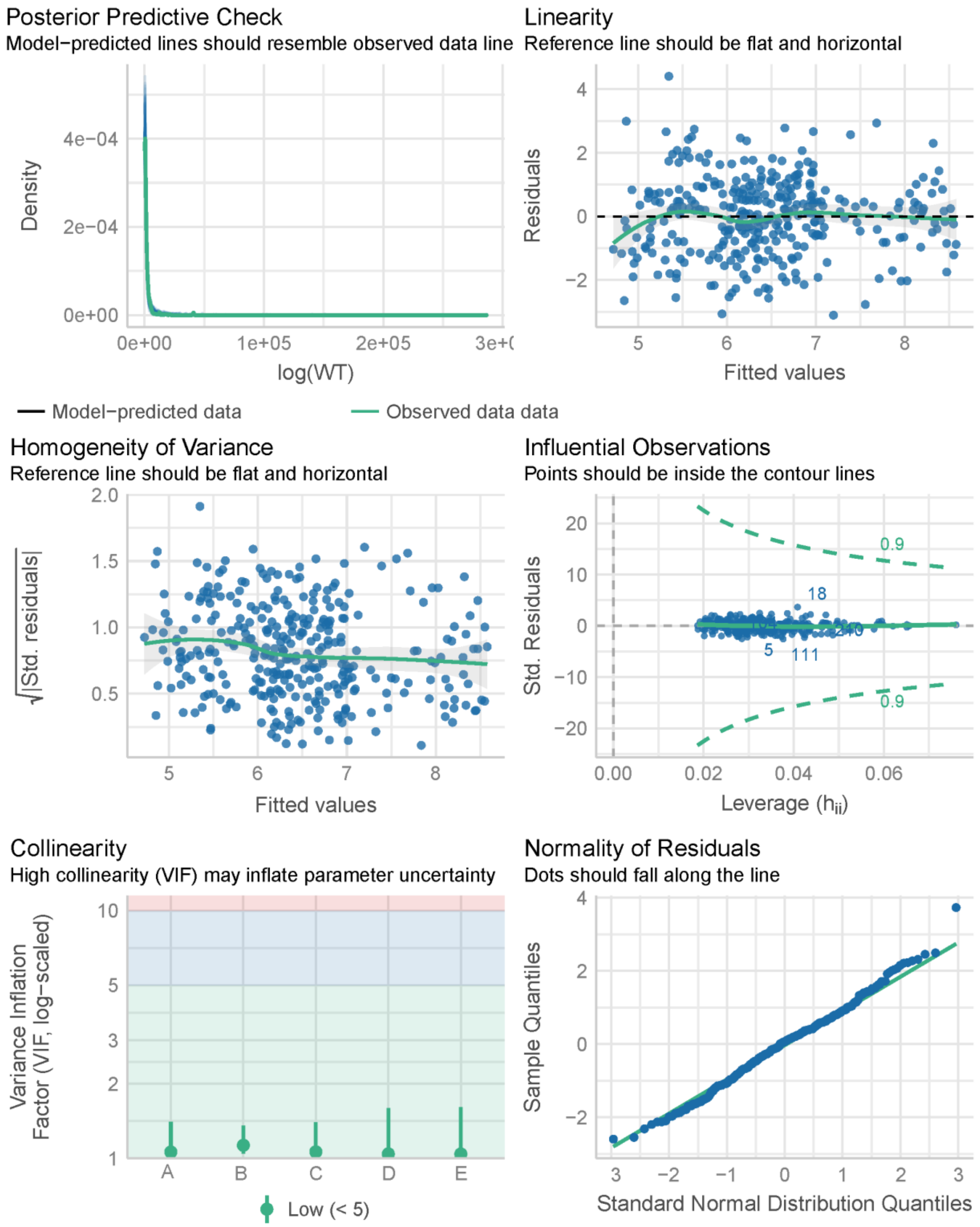

Supplementary Figure 2. Performance check of the multivariate regression model of neutralising antibody NT50 against WT after two vaccine doses in different treatment groups, adjusting for age, ethnicity, mRNA vaccine and prior infection. The variables adjusted in the regression model were labelled with the letters A to E in the Collinearity plot. Keys: A, age (per decade); B, Group (healthy control and IBD treatment groups); C, mRNA vaccine; D, prior infection; E, interval days between blood sampling and 2nd vaccine dose.

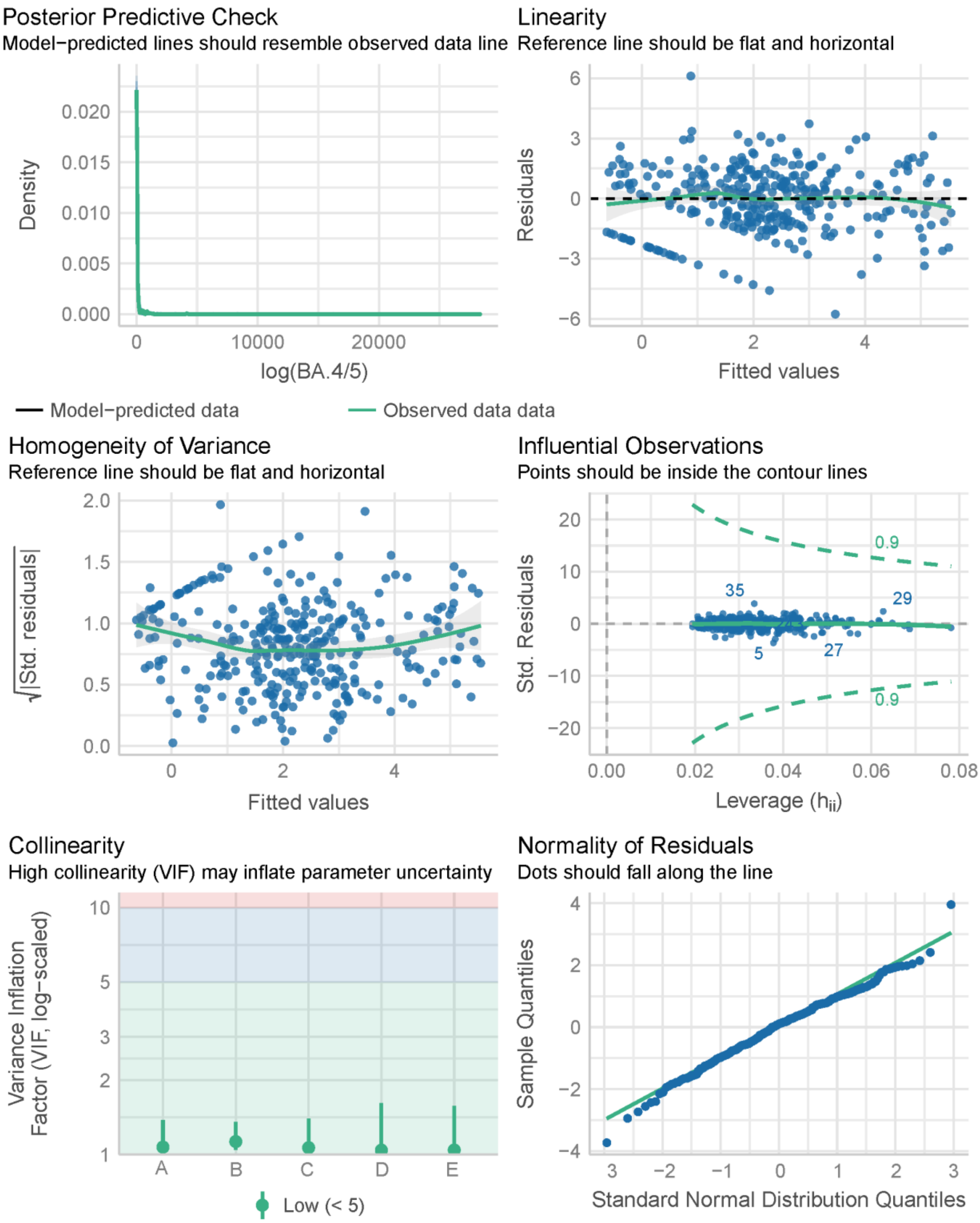

Supplementary Figure 3. Performance check of the multivariate regression model of neutralising antibody NT50 against BA.4/5 after two vaccine doses in different treatment groups, adjusting for age, ethnicity, mRNA vaccine and prior infection. The variables adjusted in the regression model were labelled with the letters A to E in the Collinearity plot. Keys: A, age (per decade); B, Group (healthy control and IBD treatment groups); C, mRNA vaccine; D, prior infection; E, interval days between blood sampling and 2nd vaccine dose.

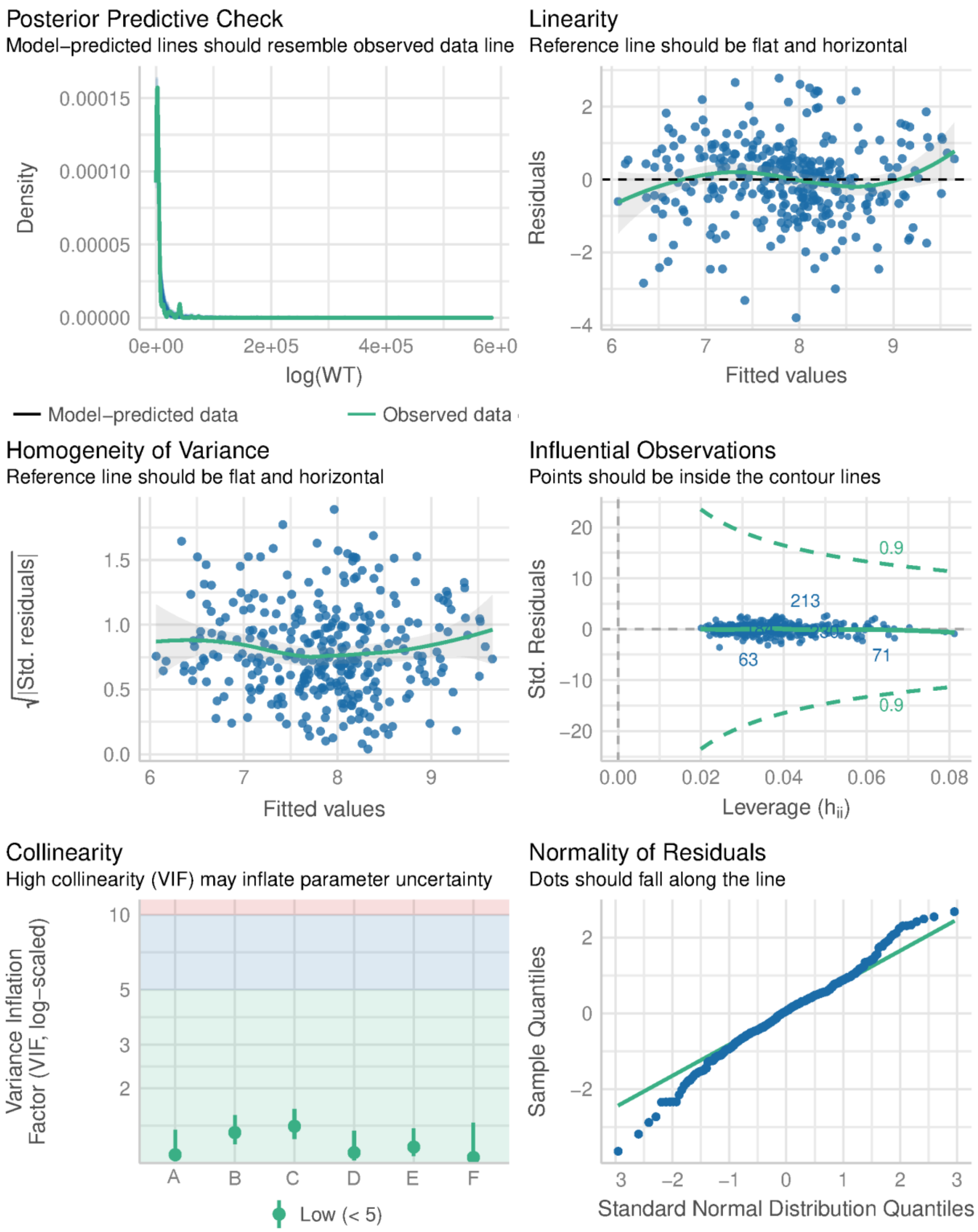

Supplementary Figure 4. Performance check of the multivariate regression model of neutralising antibody NT50 against WT after three vaccine doses in different treatment groups, adjusting for age, ethnicity, mRNA vaccine and prior infection. The variables adjusted in the regression model were labelled with the letters A to E in the Collinearity plot. Keys: A, age (per decade); B, Group (healthy control and IBD treatment groups); C, log-transformed NT50 after 2nd vaccine dose; D. homologous or heterologous

vaccine schedule; E, prior infection; F, interval days between blood sampling and 3rd vaccine dose.

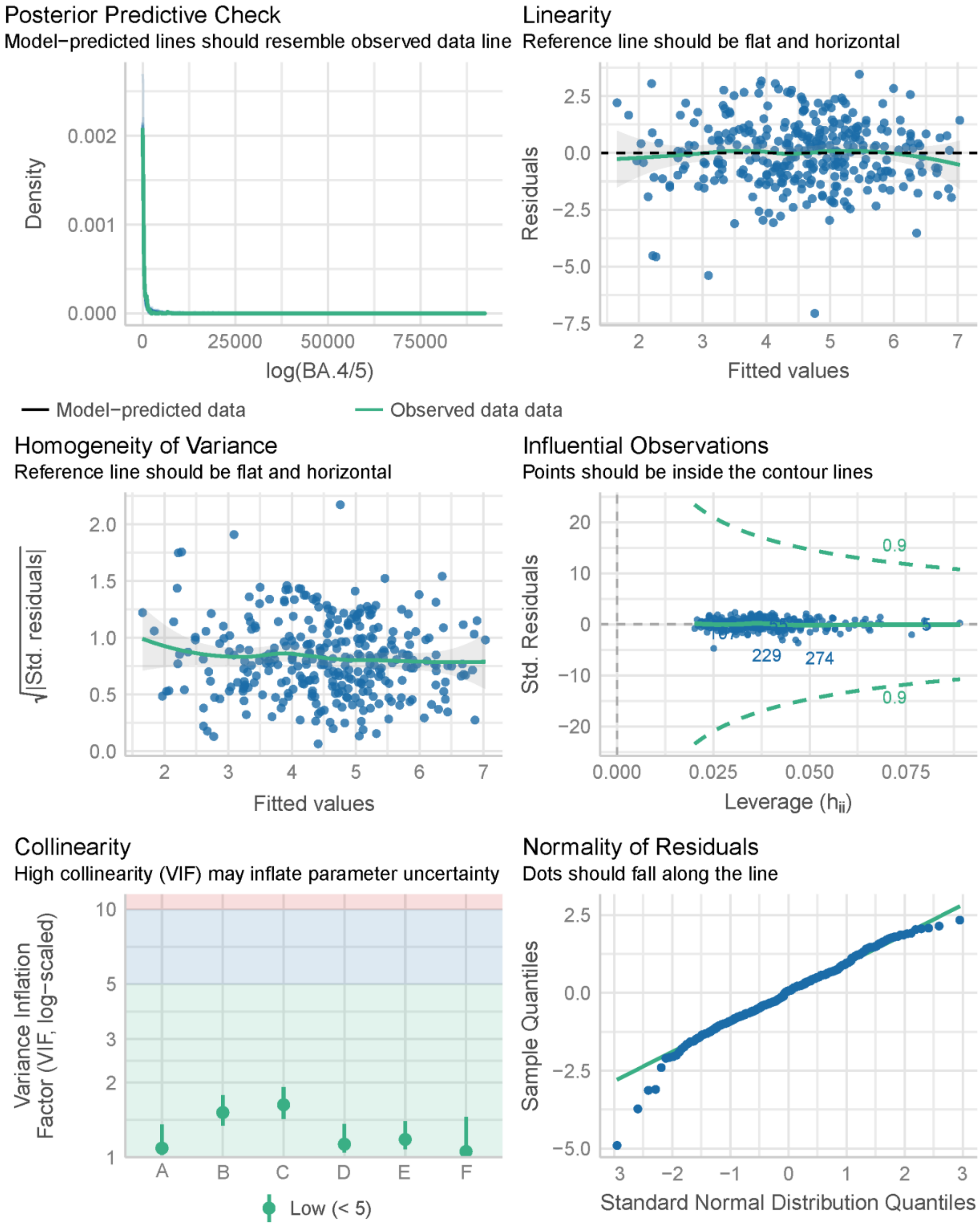

Supplementary Figure 5. Performance check of the multivariate regression model of neutralising antibody NT50 against BA.4/5 after three vaccine doses in different treatment groups, adjusting for age, ethnicity, mRNA vaccine and prior infection. The variables adjusted in the regression model were labelled with the letters A to E in the

Collinearity plot. Keys: A, age (per decade); B, Group (healthy control and IBD treatment groups); C, log-transformed NT50 after 2nd vaccine dose; D. homologous or heterologous vaccine schedule; E, prior infection; F, interval days between blood sampling and 3rd vaccine dose.

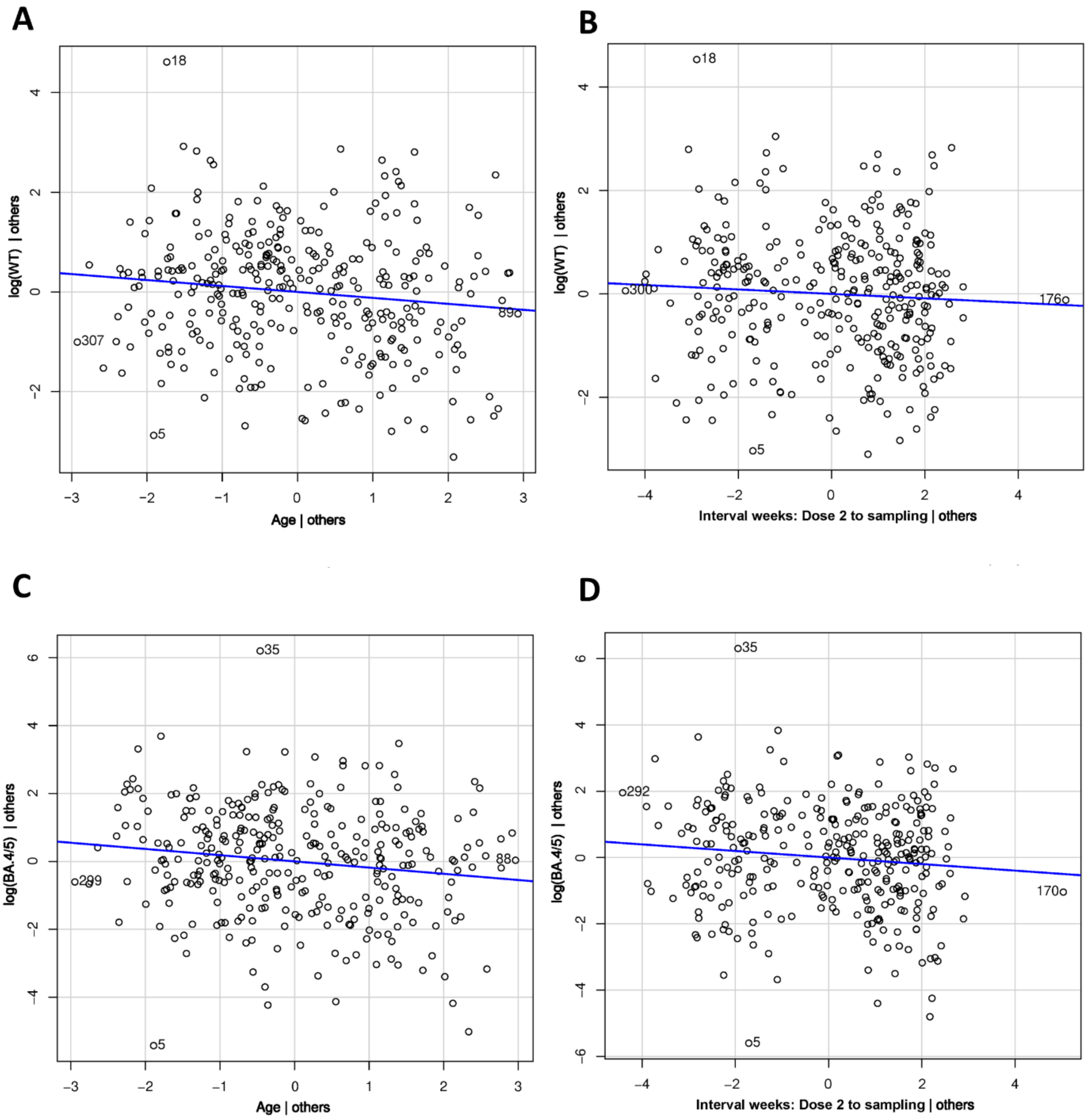

Supplementary Figure 6. Evaluating the linearity assumption in regression models with NT50 as the dependent variable after two vaccine doses using partial regression plots. A: Linear assumption partial regression plots for age in a multivariable model with NT50 against wild-type after two vaccine doses. B: Linear assumption partial regression plots for time from 2nd dose vaccination to sampling in multivariable model with NT50 against wild-type after two vaccine doses. C: Linear assumption partial regression plots for age in multivariable model with NT50 against BA.4/5 after two vaccine doses. D: Linear assumption partial regression plots for time from 2nd dose vaccination to sampling in multivariable model with NT50 against BA.4/5 after two vaccine doses.

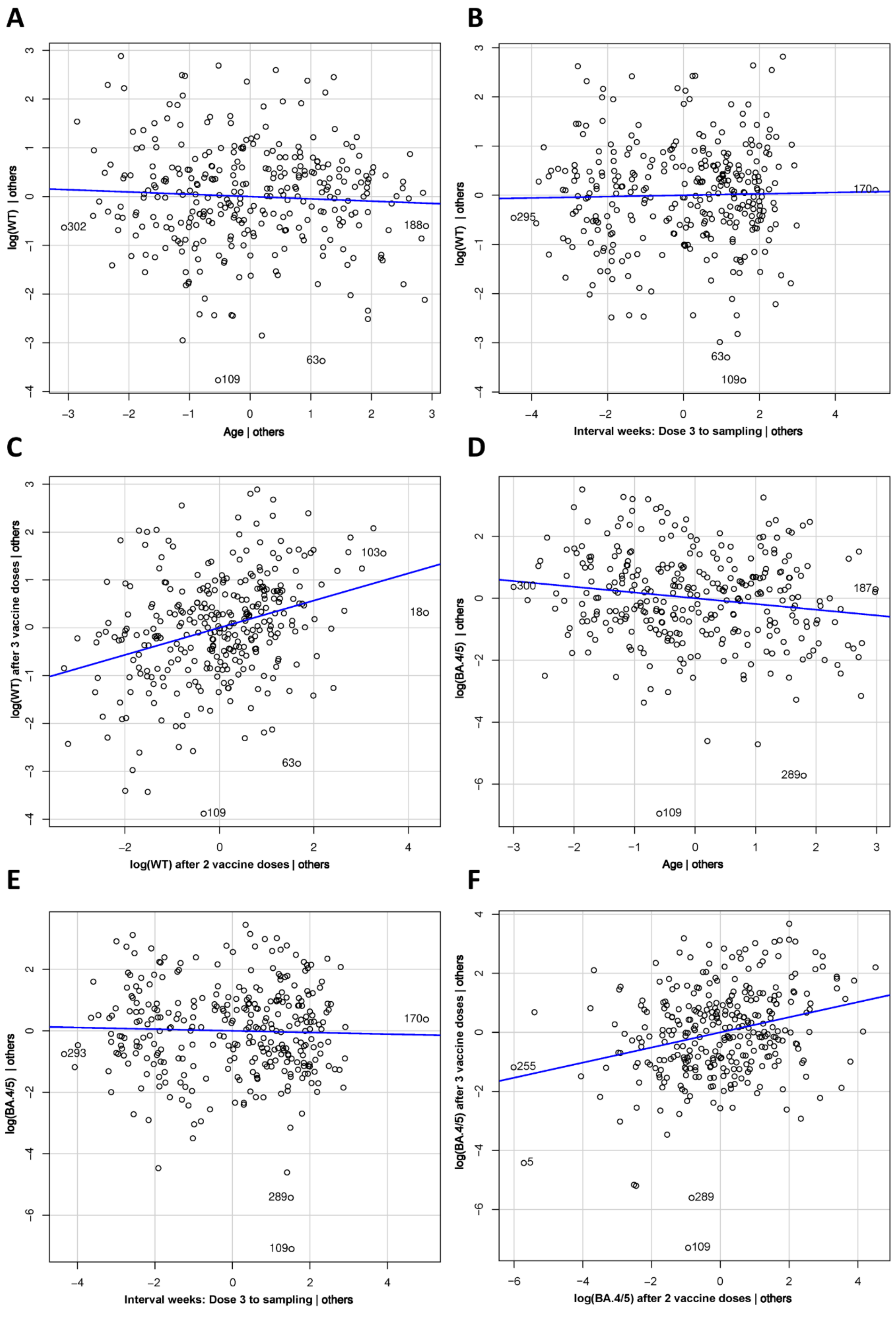

Supplementary Figure 7. Evaluating the linearity assumption in regression models with NT50 as the dependent variable after three vaccine doses using partial regression

plots. A: Linear assumption partial regression plots for age in a multivariable model with NT50 against wild-type after three vaccine doses. B: Linear assumption partial regression plots for time from 3rd dose vaccination to sampling in multivariable model with NT50 against wild-type after three vaccine doses. C: Linear assumption partial regression plots for NT50 against wild-type after 2nd dose in multivariable model with NT50 against wild-type after three vaccine doses. D: Linear assumption partial regression plots for age in multivariable model with NT50 against BA.4/5 after three vaccine doses. E: Linear assumption partial regression plots for time from 3rd dose vaccination to sampling in multivariable model with NT50 against BA.4/5 after three vaccine doses. F: Linear assumption partial regression plots for NT50 against BA.4/5 after 2nd dose in multivariable model with NT50 against BA.4/5 after three vaccine doses.

A. NT50 against wild-type after 2 vaccine doses

Variable	N	GMR (95% CI)	p
Infliximab	45/271	0.25 (0.15, 0.43)	<0.0001
Infliximab+Thiopurine	48/271	0.38 (0.22, 0.65)	0.00042
Thiopurine	63/271	0.91 (0.56, 1.49)	0.71
Tofacitinib	24/271	0.28 (0.15, 0.54)	0.00013
Ustekinumab	45/271	0.73 (0.43, 1.25)	0.26
Age (per decade)	271/271	0.91 (0.81, 1.02)	0.10
mRNA vaccine	106/271	1.36 (0.99, 1.88)	0.060
Prior infection	53/271	4.62 (3.10, 6.89)	<0.0001
Interval weeks: Dose 2 to sampling	271/271	0.95 (0.88, 1.04)	0.27

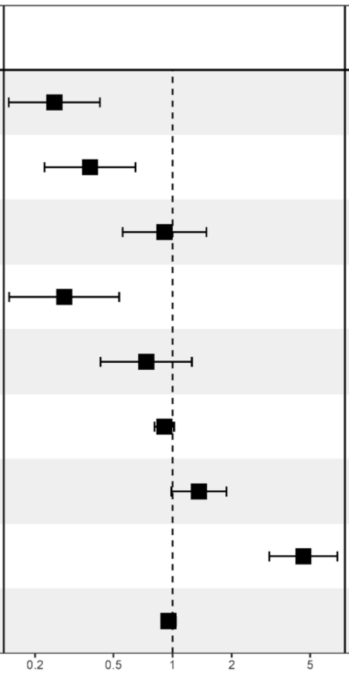

B. NT50 against BA.4/5 after 2 vaccine doses

Variable	N	GMR (95% CI)	p
Infliximab	45/271	0.16 (0.08, 0.33)	<0.0001
Infliximab+Thiopurine	48/271	0.08 (0.04, 0.17)	<0.0001
Thiopurine	63/271	0.90 (0.47, 1.75)	0.76
Tofacitinib	24/271	0.31 (0.13, 0.74)	0.0085
Ustekinumab	45/271	0.90 (0.44, 1.85)	0.77
Age (per decade)	271/271	0.80 (0.69, 0.94)	0.0074
mRNA vaccine	106/271	2.17 (1.40, 3.35)	0.00054
Prior infection	53/271	8.34 (4.85, 14.34)	<0.0001
Interval weeks: Dose 2 to sampling	271/271	0.93 (0.83, 1.04)	0.20

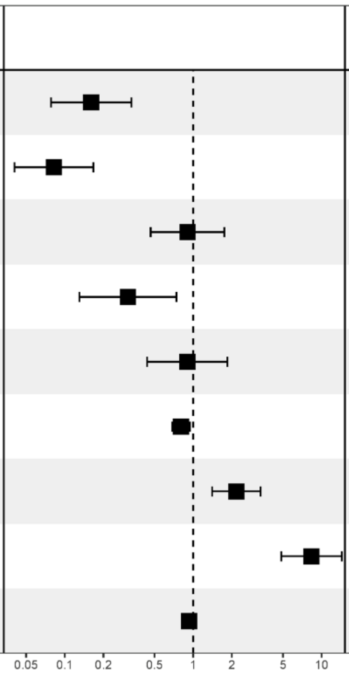

Supplementary Figure 8. Multivariable regression model for NT50 after two vaccine doses in patients with IBD (without healthy controls).

A. NT50 against wild-type after 3 vaccine doses

Variable	N	GMR (95% CI)	p
**Infliximab**	44/267	0.59 (0.36, 0.95)	0.032
**Infliximab+Thiopurine**	45/267	0.51 (0.32, 0.82)	0.0060
**Thiopurine**	61/267	0.96 (0.63, 1.47)	0.87
**Tofacitinib**	25/267	0.55 (0.31, 0.97)	0.038
**Ustekinumab**	45/267	0.91 (0.58, 1.45)	0.70
**Age (per decade)**	47/267	0.95 (0.85, 1.05)	0.27
**NT50 after two vaccine doses**	267/267	1.37 (1.24, 1.52)	<0.0001
**Prior infection**	87/267	1.60 (1.18, 2.16)	0.0026
**Interval weeks: Dose 3 to sampling**	267/267	1.01 (0.94, 1.09)	0.74
**Homologous vaccinations schedule**	107/267	1.03 (0.78, 1.37)	0.81

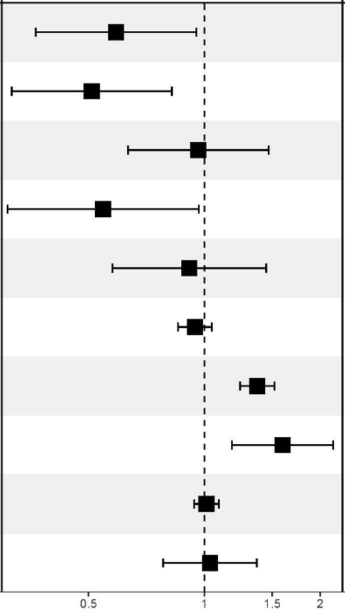

B. NT50 against BA.4/5 after 3 vaccine doses

Variable	N	GMR (95% CI)	p
**Infliximab**	44/267	0.22 (0.11, 0.44)	<0.0001
**Infliximab+Thiopurine**	45/267	0.30 (0.15, 0.62)	0.0011
**Thiopurine**	61/267	0.75 (0.42, 1.35)	0.34
**Tofacitinib**	25/267	0.50 (0.23, 1.09)	0.082
**Ustekinumab**	45/267	0.67 (0.35, 1.27)	0.22
**Age (per decade)**	47/267	0.81 (0.70, 0.94)	0.0044
**NT50 after two vaccine doses**	267/267	1.32 (1.19, 1.47)	<0.0001
**Prior infection**	87/267	1.84 (1.20, 2.82)	0.0057
**Interval weeks: Dose 3 to sampling**	267/267	0.97 (0.88, 1.08)	0.57
**Homologous vaccinations schedule**	107/267	0.94 (0.63, 1.39)	0.74

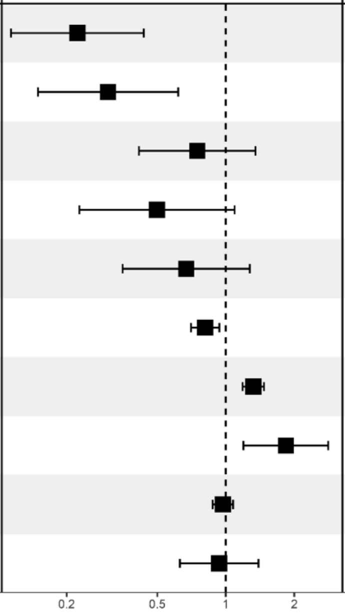

Supplementary Figure 9. Multivariable regression model for NT50 after three vaccine doses in patients with IBD (without healthy controls).

A. NT50 against wild-type after 2 vaccine doses

Variable	N	GMR (95% CI)	p
Infliximab	36/267	0.30 (0.17, 0.53)	<0.0001
Infliximab+Thiopurine	39/267	0.48 (0.28, 0.83)	0.0084
Thiopurine	51/267	1.04 (0.63, 1.74)	0.87
Tofacitinib	16/267	0.30 (0.14, 0.62)	0.0014
Ustekinumab	39/267	0.87 (0.50, 1.51)	0.62
Vedolizumab	37/267	1.33 (0.76, 2.33)	0.32
Age (per decade)	267/267	0.88 (0.78, 0.99)	0.033
mRNA vaccine	162/267	1.66 (1.20, 2.30)	0.0024
Interval weeks: Dose 2 to sampling	267/267	0.95 (0.87, 1.03)	0.23

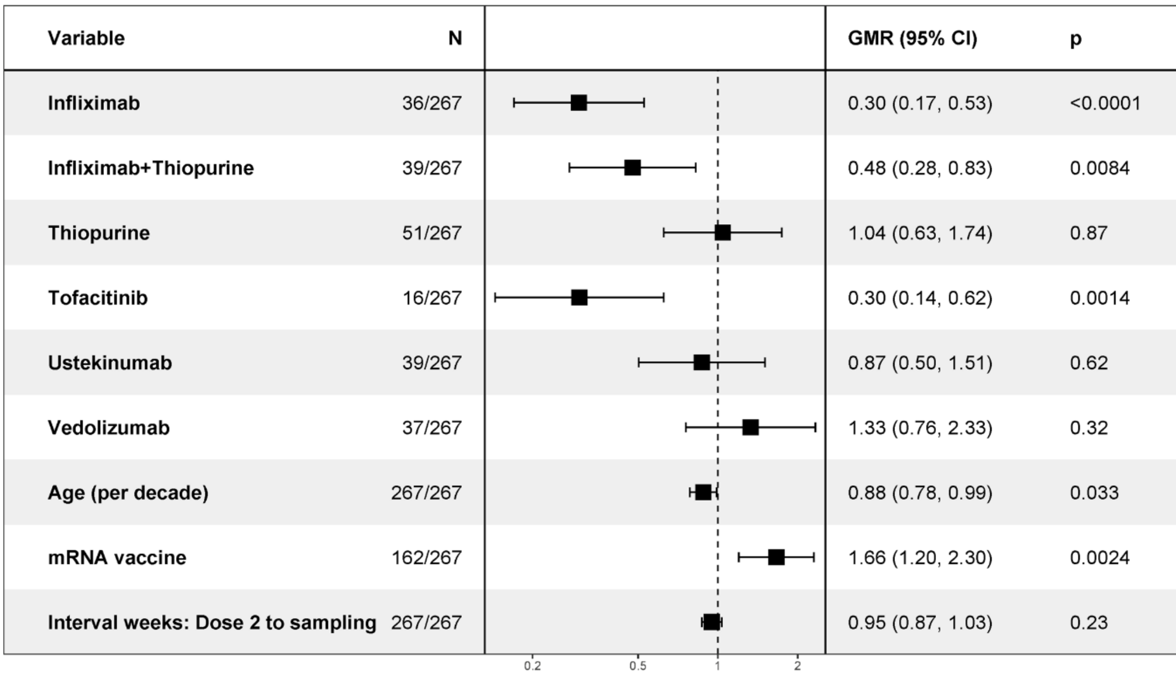

B. NT50 against BA.4/5 after 2 vaccine doses

Variable	N	GMR (95% CI)	p
Infliximab	36/267	0.22 (0.11, 0.45)	<0.0001
Infliximab+Thiopurine	39/267	0.09 (0.04, 0.17)	<0.0001
Thiopurine	51/267	0.91 (0.48, 1.71)	0.76
Tofacitinib	16/267	0.33 (0.14, 0.82)	0.017
Ustekinumab	39/267	1.14 (0.58, 2.24)	0.71
Vedolizumab	37/267	1.34 (0.68, 2.64)	0.40
Age (per decade)	267/267	0.80 (0.69, 0.93)	0.0028
mRNA vaccine	162/267	2.75 (1.84, 4.11)	<0.0001
Interval weeks: Dose 2 to sampling	267/267	0.90 (0.81, 1.01)	0.065

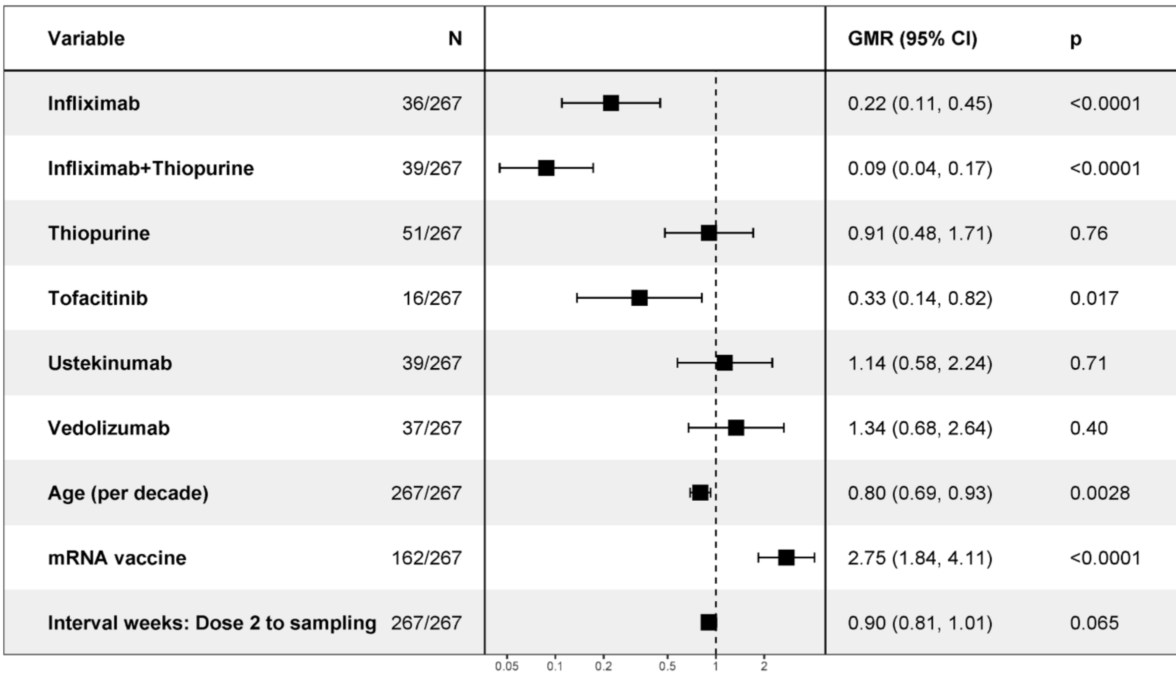

Supplementary Figure 10. Multivariable regression model for NT50 after two vaccine doses in participants without prior infection.

A. NT50 against wild-type after 3 vaccine doses

Variable	N	GMR (95% CI)	p
Infliximab	31/222	0.66 (0.40, 1.09)	0.10
Infliximab+Thiopurine	28/222	0.47 (0.28, 0.78)	0.0039
Thiopurine	42/222	0.88 (0.56, 1.37)	0.56
Tofacitinib	14/222	0.36 (0.19, 0.70)	0.0027
Ustekinumab	31/222	0.88 (0.54, 1.42)	0.60
Vedolizumab	34/222	0.99 (0.62, 1.60)	0.98
Age (per decade)	222/222	1.02 (0.91, 1.13)	0.77
NT50 after 2 vaccine doses	222/222	1.36 (1.21, 1.51)	<0.0001
Interval weeks: Dose 3 to sampling	222/222	0.98 (0.90, 1.06)	0.56
Homologous vaccine schedule	132/222	0.80 (0.59, 1.07)	0.14

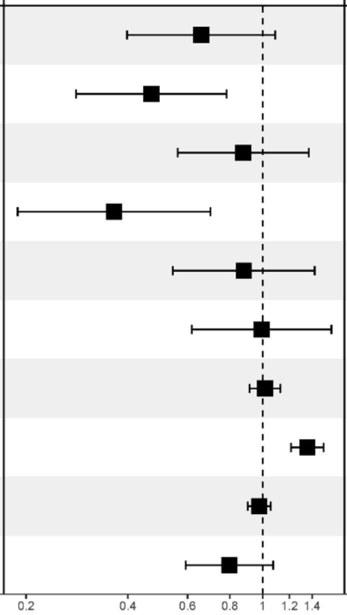

B. NT50 against BA.4/5 after 3 vaccine doses

Variable	N	GMR (95% CI)	p
Infliximab	31/222	0.39 (0.19, 0.81)	0.012
Infliximab+Thiopurine	28/222	0.26 (0.11, 0.58)	0.0011
Thiopurine	42/222	0.72 (0.38, 1.38)	0.32
Tofacitinib	14/222	0.48 (0.19, 1.25)	0.13
Ustekinumab	31/222	0.62 (0.31, 1.26)	0.19
Vedolizumab	34/222	0.97 (0.49, 1.91)	0.92
Age (per decade)	222/222	0.84 (0.72, 0.98)	0.030
NT50 after 2 vaccine doses	222/222	1.46 (1.28, 1.67)	<0.0001
Interval weeks: Dose 3 to sampling	222/222	0.99 (0.88, 1.10)	0.81
Homologous vaccine schedule	132/222	0.71 (0.45, 1.10)	0.13

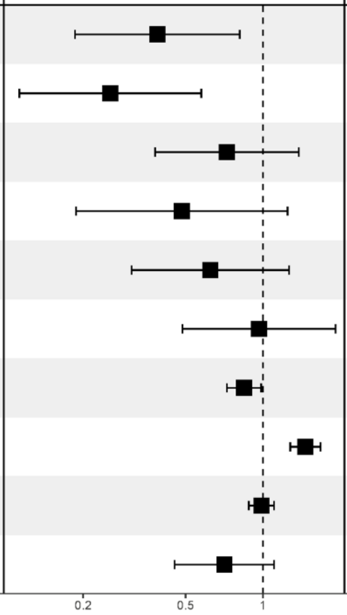

Supplementary Figure 11. Multivariable regression model for NT50 after three vaccine doses in participants without prior infection.

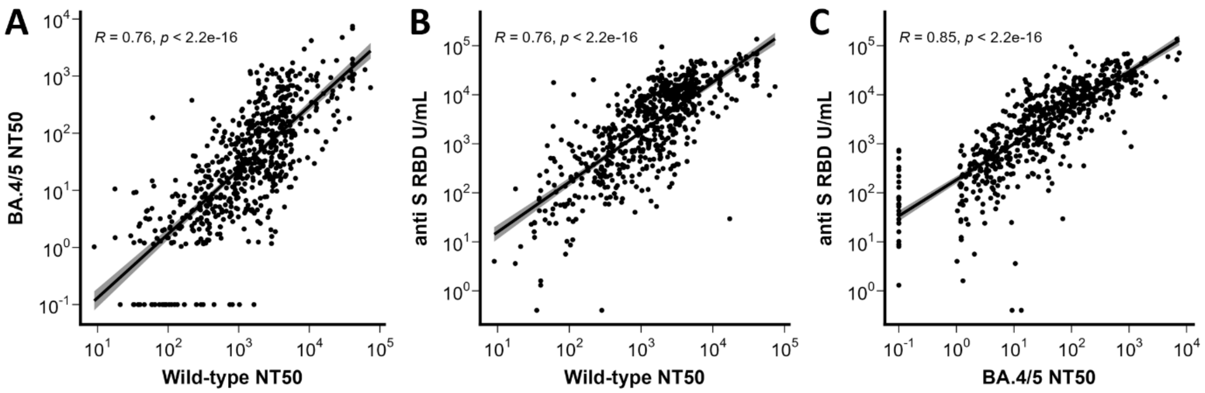

Supplementary Figure 12. Spearman correlation of NT50 against wild-type and BA.4/5 (A), NT50 against wild-type and anti-Spike RBD (B), and NT50 against BA.4/5 and anti-Spike RBD (C).

Supplementary Table 1. The VIP Consortium Member Lists

First Name	Surname
Ijeoma	Chukwurah
Sulaimaan	Haq
Jonathan	Lo
Parita	Shah
Stephanie	Wilken-Smith
Anitha	Ramanathan
Mikin	Patel
Lidia	Romanczuk
Rebecca	King
Jason	Domingo
Djamila	Shamtally
Vivien	Mendoza
Joanne	Sanchez
Hannah	Stark
Bridget	Knight
Louise	Bee
Charmaine	Estember
Anna	Barnes
Darcy	Watkins
Sam	Stone
John	Kirkwood
Marian	Parkinson
Helen	Gardner-Thorpe
Kate	Covil

Lauranne	Derikx
Beatriz	Gros Alcalde
Irish	Lee
Bessie	Cipriano
Giuseppe	Ruocco
Manisha	Baden
Graham	Cooke
Evgenia	Kourampa
Ciro	Pasquale
Elena	Robisco-Diaz
Suhaylah	Bhatti
